# T2SS Components GspJ, GspL, and GspM Regulate Virulence in *Vibrio alginolyticus*

**DOI:** 10.3390/ani16193121

**Published:** 2026-10-05

**Authors:** Le Zhang, Qiang Chen, Xiaojin Xu, Chunmei Zhang, Xufei Liu, Chengcheng Li, Lunrui Zhang, Yujin Xiao, Yuxuan Sha, Guiling Zhang, Yunong Chen, Pocheng Chen

**Affiliations:** 1 Fisheries College, Jimei University, Xiamen 361021, China; 18116977662@163.com (L.Z.); ahcq@jmu.edu.cn (Q.C.);; 2 State Key Laboratory of Mariculture Breeding, Fujian Provincial Key Laboratory of Marine Fishery Resources and Eco-Environment, Fisheries College, Jimei University, Xiamen 361021, China; 3 The School of Foreign Languages Studies, Jimei University, Xiamen 361021, China

**Keywords:** *Vibrio alginolyticus*, *gspJ*, *gspL*, *gspM*, RNA interference, virulence, label-free proteomic analysis, *Epinephelus coioides*

## Abstract

*Vibrio alginolyticus* is a major aquaculture pathogen whose virulence is largely mediated by the type II secretion system (T2SS). However, the functions of key T2SS components, including *gspJ*, *gspL*, and *gspM*, remain unclear. Earlier research indicated that a *Scutellaria baicalensis* extract at 8 mg/mL markedly suppressed the growth, motility, and biofilm-forming capacity of *V. alginolyticus* ND-01 (OD_600_ = 0.5) following 12 h of treatment, with the resulting stress substantially altering the expression of multiple genes, including *gspJ*, *gspL*, and *gspM*. The *gspJ*-RNAi, *gspL*-RNAi, and *gspM*-RNAi strains were constructed using RNAi. Biological functional assays demonstrated that stable silencing of *gspJ*, *gspL*, and *gspM* markedly decreased biofilm formation ability (84.01%, 24.99%, and 78.26%, respectively) and host cell adhesion (73.57%, 75.65%, and 79.61%, respectively); *gspJ* and *gspM* silencing reduced motility (54.82% and 57.42%, respectively), whereas *gspL* silencing enhanced motility by 38.49%, indicating their essential roles in virulence-associated phenotypes. Notably, in vivo infection of *Epinephelus coioides* revealed that *gspL*-RNAi significantly attenuated pathogenicity, as evidenced by reduced host mortality (50%) and decreased bacterial colonization in liver and spleen tissues, compared with the wild-type group. Consistently, Label-Free Proteomic Analysis of the *gspL*-RNAi strain identified 189 differentially expressed proteins in the *gspL*-silenced strain, with enrichment in two-component signaling, ABC transport systems, and amino acid metabolism. Network analysis further positioned *gspL* as a central regulatory hub linking T2SS activity to signal transduction, transport, and metabolic pathways. In addition, the expression of *gspJ*-RNAi, *gspL*-RNAi, and *gspM*-RNAi exhibited distinct responses to environmental stresses, including pH and temperature variation. Collectively, our findings highlight that *gspJ*, *gspL*, and *gspM* are critical regulators of virulence in *V. alginolyticus*, with *gspL* playing a central role in coordinating T2SS function with global regulatory networks. This study advances our understanding of the pathogenic mechanisms of *V. alginolyticus* and provides potential targets for the development of anti-virulence aquaculture strategies.

## Simple Summary

*Vibrio alginolyticus* causes serious infections in farmed fish. Its ability to cause disease relies heavily on a protein secretion system called T2SS. In this study, we focused on three T2SS-related genes: *gspJ*, *gspL*, and *gspM*. When these genes were silenced, the bacterium formed less biofilm, moved less, and attached to host cells less effectively; also, silencing *gspL* significantly reduced death rates. Protein analysis showed that *gspL* acts as a central regulator, connecting the secretion system to other important bacterial processes. Our results show that *gspJ*, *gspL*, and *gspM* may serve as promising targets for new strategies to control infections in aquaculture.

## 1. Introduction

Bacteria of the genus Vibrio are widely distributed in marine and estuarine environments and include numerous pathogenic and opportunistic species of importance to aquaculture. Vibriosis is one of the predominant bacterial diseases affecting cultured fish, and disease outbreaks can result in substantial mortality and economic losses. Among these pathogens, *Vibrio alginolyticus* is a Gram-negative halophilic bacterium that has emerged as one of the most significant opportunistic pathogens in marine aquaculture systems worldwide [1,2]. The pathogen is widely distributed in coastal and estuarine environments, where it poses a continuous threat to a variety of commercially important aquatic species [3]. Severe infections caused by *V. alginolyticus* have been documented in finfish, including orange-spotted grouper (*Epinephelus coioides*), large yellow croaker (*Larimichthys crocea*), and Japanese seabass (*Lateolabrax japonicus*), as well as in crustaceans and mollusks [1,2,4,5]. Disease outbreaks often lead to high mortality rates, with septicemia, skin ulceration, and extensive tissue damage being characteristic clinical signs, leading to considerable economic losses for the aquaculture industry [4].

The infectivity of *V. alginolyticus* is mediated by multiple virulence-associated mechanisms, within which the type II secretion system (T2SS) is important for the extracellular transport of proteins involved in bacterial survival, colonization, and host interactions [6,7]. The T2SS is a multiprotein complex composed of an outer membrane secretin, an inner membrane assembly platform, a cytoplasmic ATPase, and a pseudopilus. Within this machinery, GspJ is a minor pseudopilin associated with the pseudopilus, whereas GspL and GspM are components of the inner membrane assembly platform [8,9]. GspL interacts with the secretion ATPase GspE and contributes to coupling ATP-dependent energy to pseudopilus assembly, while GspM interacts with other membrane components and contributes to the organization and stability of the secretion machinery. These coordinated structural components are therefore positioned to influence the secretion process and, consequently, virulence-associated functions. Although the structural mechanisms of the T2SS are increasingly well understood, the specific virulence functions of its core components, including gspJ, gspL, and gspM, remain largely uncharacterized [10].

Environmental conditions are also important determinants of Vibrio physiology and pathogenicity. Changes in temperature and pH can alter bacterial stress responses and the expression of virulence-associated genes in *V. alginolyticus*. Previous studies have shown that temperature can modulate virulence-related regulatory pathways, while pH and other environmental stresses can influence genes associated with adhesion, motility, and chemotaxis. However, whether the T2SS components *gspJ*, *gspL*, and *gspM* exhibit similar transcriptional responses to environmental variation remains unclear. Characterizing their responses to temperature and pH may therefore provide insight into how T2SS-associated functions are regulated under changing aquaculture conditions.

Previous studies from our laboratory showed that *S. baicalensis* extract inhibited the virulence-associated phenotypes of *V. alginolyticus* and altered the expression of several genes, including *gspJ*, *gspL*, and *gspM.* These observations prompted us to investigate whether these T2SS-associated genes contribute directly to the virulence-related characteristics of *V. alginolyticus* [11].

Based on the structural roles of these proteins within the T2SS and our previous observations, we hypothesized that *gspJ*, *gspL*, and *gspM* contribute to the virulence-associated functions of *V. alginolyticus* and that suppression of these genes would impair phenotypes related to pathogenicity, including biofilm formation, motility, and host adhesion. We further predicted that the transcription of these genes would respond differently to changes in environmental temperature and pH.

At present, the management of *V. alginolyticus* infections in aquaculture depends largely on antibiotics and chemical disinfectants. Nevertheless, the overuse of these substances has resulted in the development of antimicrobial resistance, environmental pollution, and issues related to food safety [12,13]. Thus, eco-sustainable and precisely targeted strategies for disease control, founded on a deeper insight into the pathogenic processes of bacteria, are urgently required. RNA interference (RNAi) technology provides a powerful tool for investigating gene function and has been successfully employed to elucidate virulence mechanisms in various bacterial pathogens [14].

In this study, we constructed stable, RNAi-silenced strains targeting *gspJ*, *gspL*, and *gspM*. A comprehensive evaluation was performed to determine the effects of gene silencing on bacterial proliferation, biofilm-forming capability, adherence, chemotaxis, and motility, in addition to in vivo virulence, with *E. coioides* serving as the model host. Moreover, label-free proteomic characterization of the *gspL*-RNAi strain was undertaken to reveal downstream target proteins and regulatory cascades associated with virulence. We further analyzed the expression profiles of *gspJ, gspL*, and *gspM* under diverse environmental settings, such as pH and temperature, to shed light on their roles in environmental adaptation. We examined how silencing *gspJ*, *gspL*, and *gspM* influences the susceptibility of *V. alginolyticus* to the extract of *S. baicalensis*. Our findings indicate that the core components of the T2SS contribute to the virulence of *V. alginolyticus*, thereby supporting the development of novel targeted strategies for disease prevention and control in aquaculture.

## 2. Materials and Methods

### 2.1. Bacterial Isolates and Cultivation

The bacterial strains and fish used in this study were obtained as follows. The wild-type *V. alginolyticus* (ND-01), previously obtained and characterized from an infected E. coioides, was stored at −80 °C in our laboratory [15]. *V. alginolyticus* (ND-01) was spread onto LB agar plates and incubated overnight at 28 °C. Subsequently, individual colonies were transferred into 1.5 mL of LB broth and cultivated at 220 rpm in a shaking incubator at 28 °C for 6 h. *Escherichia coli* DH5α competent cells (Beijing Quanshijin Biotechnology Co., Ltd., Beijing, China) and *E. coli* SM10 (Beijing TianGen Biological Co., Ltd. Beijing, China) were used for recombinant plasmid construction and conjugation, respectively; DH5α and SM10 were streaked on LB agar in the same way and cultured at 37 °C for 12 h. Subsequently, they were inoculated into LB liquid medium and cultured at the same temperature and shaking rate for another 6 h, and they were also stored at −80 °C. Healthy *E. coioides* were purchased from a commercial farm in Zhangpu, Fujian Province, and acclimated in laboratory tanks prior to infection experiments.

### 2.2. Validation of gspJ, gspL, and gspM Expression Levels

To validate prior transcriptomic data indicating significant downregulation of gspJ, gspM, and gspL in *V. alginolyticus* treated with S. baicalensis extract, quantitative real-time PCR (qRT-PCR) was performed using the standard curve method. The wild-type *V. alginolyticus* was cultured overnight in LB broth at 28 °C with shaking, followed by treatment with 70 mg/mL *S. baicalensis* extract for 4 h. Total RNA was extracted using TransZol Up reagent (Beijing Transgen Biological Co., Ltd, China) according to the manufacturer’s instructions, including DNase I treatment. RNA quality and concentration were assessed, and cDNA was synthesized using TransScript First-Strand cDNA Synthesis SuperMix. qRT-PCR was performed on a QuantStudio 6 Flex system using TransStart Top Green qPCR SuperMix, each with three technical replicates [15]. The *gyrB* gene was used as an internal control, and relative gene expression levels were calculated using the 2^−ΔΔCT^ method. Primer sequences are listed in Table 1.

### 2.3. Sequence Alignment and Domain Prediction

Nucleotide sequences of *gspJ*, *gspM*, and *gspL* were retrieved from the *V. alginolyticus* ND-01 genome database. Sequence alignment was performed using BLAST (BLAST+ 2.17.0) against the NCBI non-redundant database. Multiple sequence alignment was conducted using Clustal X 1.8 with default parameters. Phylogenetic trees were constructed using the neighbor-joining method in MEGA 7.0 with 1000 bootstrap replicates. Protein domains were predicted using the InterPro database, and conserved domains were visualized using IBS Illustrator (IBS 2.0).

### 2.4. Protein Homology Modeling

Amino acid sequences were translated from the nucleotide sequences of the target genes. Three-dimensional structural models of the target proteins were constructed using SWISS-MODEL. The predicted structures were further refined by docking template ligands onto the models using PyMOL 3.1.

### 2.5. Construction of gspJ, gspL, and gspM Stable, Silenced Strains

Stable gene silencing of gspJ, gspL, and gspM in *V. alginolyticus* and *E. coli* SM10 handling were conducted as described previously [16]. To construct stable, silenced strains targeting *gspJ*, *gspL*, and *gspM* in *V. alginolyticus*, an antisense RNA strategy using the pACYC184 plasmid was employed. The plasmid was extracted from *E. coli* using the EasyPure Plasmid MiniPrep Kit, linearized by double digestion with BamHI and SphI, and the 4200 bp fragment was purified using the EasyPure Quick Gel Extraction Kit. Three pairs of shRNAs were designed for each gene with the aid of the Thermo Fisher RNAi Designer online tool (http://rnaidesigner.thermofisher.com accessed on 25 August 2026), followed by construction at Bright Gene Co., Ltd., Shanghai, China (Table 2), synthesized, annealed, and ligated into the linearized vector using T4 DNA ligase. Recombinant plasmids were introduced into *E. coli* DH5α, and positive clones were confirmed by sequencing. For conjugation, recombinant plasmids were introduced into *E. coli* SM10 competent cells via heat shock. Donor (*E. coli* SM10) and recipient (wild-type *V. alginolyticus*) cells were mixed at optimized ratios, spotted onto LB agar plates, and incubated overnight at 28 °C. Transconjugants were selected on TCBS agar supplemented with chloramphenicol, and the presence of recombinant plasmids was confirmed by sequencing. Silencing efficiency was assessed by qRT-PCR, with the *V. alginolyticus* strain containing an empty plasmid used as the control. Total RNA was extracted from the stably silenced strains and the empty-plasmid control. Using *gyrB* as the internal reference gene, relative expression levels of *gspJ*, *gspL*, and *gspM* were calculated using the 2^−ΔΔCT^ method, confirming successful silencing of *gspJ*, *gspL*, and *gspM*.

### 2.6. Growth Curve Determination

Growth curves were compared among the wild-type, *phoB*-RNAi strain, and *phoR*-RNAi strains of *V. alginolyticus*. Wild-type *V. alginolyticus*, *gspJ*-RNAi, *gspL*-RNAi, and *gspM*-RNAi strains were cultured overnight in LB medium at 28 °C with shaking at 220 rpm. Chloramphenicol was added to the medium for the silenced strains. Overnight cultures were subcultured to an OD_600_ of 0.3–0.4, adjusted to an OD_600_ value of 0.2, and then diluted 1000-fold. Aliquots (200 μL) of each diluted suspension were transferred into a 96-well microplate, with eight replicates per group. Wild-type *V. alginolyticus* carrying the empty plasmid served as the control. The plate was incubated at 28 °C; then, OD_600_ was monitored at 30 min intervals for 48 h.

### 2.7. Biofilm Formation Assay

Biofilm formation capability was assessed using the crystal violet staining method [16]. Briefly, overnight cultures of the wild-type strain, together with *gspJ*-RNAi, *gspL*-RNAi, and *gspM*-RNAi strains were grown at 28 °C and 220 rpm in LB medium, respectively. After adjusting the bacterial concentration to an OD_600_ of 0.2 with PBS, to each well of a 96-well plate, 100 μL of bacterial suspension was added. Plates were incubated at 28 °C for 24 h. All samples were tested in triplicate. The bacterial suspension was then carefully aspirated, and the wells were gently washed with PBS (two to three times) to remove non-adherent bacteria, followed by drying at 65 °C for 75 min. The attached biofilms were fixed and stained with 150 μL of 0.2% crystal violet solution (Shanghai criac.org Co., Shanghai, China) for a period of 12 min; subsequently, the staining solution was removed. The crystal violet incorporated into the biofilm was thoroughly solubilized by gentle, repetitive pipetting and vortexing to ensure complete mixing. After the dye had been fully dissolved, the assay was resumed. The wells were rinsed four to six times with PBS until the effluent was clear and the plates were air-dried. Then, each well received 170 μL of 45% acetic acid (Beijing Lablead Biotechnology Co., Beijing, China). The dye bound to the biofilms was measured at 590 nm using a microplate reader. After the biofilm was fully dissolved, the OD_590_ values of the wild-type, *gspJ*-RNAi, *gspL*-RNAi, and *gspM*-RNAi strains of *V. alginolyticus* were measured, with sterile LB liquid medium as the control. The entire experiment was repeated three times independently.

### 2.8. Motility Assay

Motility assay was assessed on LB soft agar plates (0.5% agar concentration; Beijing Lablead Biotechnology Co.). Overnight cultures of wild-type *V. alginolyticus* and *gspJ*-RNAi, *gspL*-RNAi, and *gspM*-RNAi strains (with chloramphenicol added for silenced strains) were subcultured to OD_600_ = 0.2. Each suspension (1 μL) was spotted onto the center of the plates and air-dried for 30 min [16]. Plates were incubated undisturbed at 28 °C for 12–16 h, and swimming diameters were measured using a colony counter. Three replicates were performed per group.

### 2.9. Adhesion Assay

The adhesion ability of *V. alginolyticus* strains to host surfaces was assessed using skin mucus collected from *E. coioides*. Mucus was collected by gentle scraping, centrifuged at 2000 × *g* for 30 min at 4 °C, and sequentially filter-sterilized via 0.45 μm and 0.22 μm membranes. Protein concentration was determined using the Bradford assay and adjusted to a final concentration of 1 mg/mL with sterile PBS. Aliquots were stored at −80 °C until use. For adhesion assays, sterile glass slides were coated with 20 μL of standardized mucus and air-dried overnight. Bacterial cultures grown to the mid-log phase were adjusted to OD_600_ = 0.3. Mucus-coated slides were fixed with 4% paraformaldehyde for 30 min, followed by the addition of 200 μL of bacterial suspension, and cultured at 28 °C in a humidified chamber for 2 h. After incubation, slides were washed three times using PBS to eliminate non-adherent bacteria. Adherent bacteria were fixed with 4% paraformaldehyde for 25 min, stained with 0.1% crystal violet for 3 min, and visualized under a light microscope at 1000× magnification. Bacterial cells were counted in 15 randomly selected fields per slide. All assays were performed in triplicate.

### 2.10. Chemotaxis Assay

To quantify bacterial chemotaxis toward mucus, a capillary assay was performed. Overnight cultures of wild-type *V. alginolyticus* and *gspJ*-RNAi, *gspL*-RNAi, and *gspM*-RNAi strains were adjusted to OD_600_ = 1.0 with sterile PBS. Glass capillaries filled with sterile mucus were sealed at one end, and the open end was placed in a syringe containing 0.25 mL of bacterial suspension. The assembly was incubated horizontally at 28 °C for 1 h. After incubation, the capillary contents were expelled into PBS, and ten-fold serial dilutions (10^−3^ to 10^−7^) were plated onto LB agar. Colonies were counted after incubation at 28 °C for 12 h. Three replicates were performed for each dilution.

### 2.11. Expression of gspJ, gspL, and gspM Under Different pH Conditions

To examine the effect of pH on gene expression, wild-type *V. alginolyticus* was cultured to the mid-log phase (OD_600_ = 0.3–0.4) and then transferred to LB medium adjusted to pH 5, 6, 7, 8, or 9. After 6 h of incubation, total RNA was extracted, and gene expression was analyzed by qRT-PCR as described in Section 2.2.

### 2.12. Expression of gspJ, gspL, and gspM Under Different Temperature Conditions

Wild-type *V. alginolyticus* was cultivated in LB medium at 28 °C with shaking at 220 rpm to an OD_600_ of 0.3–0.4. Aliquots of the cultures were subsequently exposed to 4, 15, 28, 37, and 44 °C for 3 h. Total RNA was extracted, and qRT-PCR was performed to analyze gene expression.

### 2.13. Label-Free Proteomic Analysis

Proteins of wild-type *V. alginolyticus* and the *gspL*-RNAi strain were purified using the SDT lysis method and quantified using a BCA assay. Following LC–MS/MS analysis, raw data files were processed using MaxQuant software (version 1.5.3.17) for database searching, protein identification, and quantification [7]. Quantitative data for the target protein set were normalized, and proteins were classified across both sample and expression dimensions [8]. Subcellular localization was predicted using CELLO, protein domains were analyzed using the Pfam database, and Gene Ontology (GO) annotation was performed using Blast2GO 4.1 (https://www.blast2go.com/ accessed on 25 August 2026) [9]. KEGG pathway annotation was conducted using KAAS (https://www.genome.jp/tools/kaas/ accessed on 25 August 2026). Enrichment analysis was performed using Fisher’s exact test [10] to compare the distribution of GO terms, KEGG pathways, and protein domains between the target protein set and the overall proteome [11].

### 2.14. Virulence Assessment of Wild-Type V. alginolyticus and the gspL-RNAi Strain

To assess the virulence of the *gspL*-RNAi strain relative to wild-type *V. alginolyticus*, an in vivo infection model was established using *E. coioides* (15.2 ± 0.5 cm; 48.2 ± 3.4 g) acclimated at 28 °C for one week and randomly divided into three groups (20 fish per group): negative control (sterile PBS), positive control (wild-type *V. alginolyticus*), and experimental group (*gspL*-RNAi strain). Bacterial strains were cultured to the logarithmic phase, adjusted to 1.0 × 10^8^ CFU/mL in PBS, and maintained on ice. Each fish received an intraperitoneal injection of 200 μL of the corresponding suspension. Mortality was monitored daily for 14 days.

For bacterial load quantification, a separate experiment was conducted with 60 fish per group. On days 1–6 post-infection, three fish per group were sacrificed, and liver and spleen tissues were collected. Total DNA was extracted, and bacterial burden was quantified by qPCR using *V. alginolyticus gyrB*-specific primers, as described in Section 2.2.

### 2.15. Antibacterial Assay

To assess antibacterial activity, wild-type *V. alginolyticus* and *gspJ*-RNAi, *gspL*-RNAi, and *gspM*-RNAi strains were cultured overnight, adjusted to 1.0 × 10^8^ CFU/mL, and spread onto LB agar plates.

Wells (6 mm in diameter) were punched into the agar and filled with 20 μL of *S. baicalensis* extract (70 mg/mL). After cultivation at 28 °C for 24 h, inhibition zone diameters were measured. Sterile distilled water was employed as the negative control. All experiments were performed in triplicate for each group.

### 2.16. Data Analysis

IBM SPSS Statistics 26.0 was used for statistical analyses. All data are expressed as mean ± standard deviation (SD) from at least three independent biological replicates. One-way analysis of variance (ANOVA) was used for multiple group comparisons, followed by Tukey’s honest significant difference (HSD) post-hoc test. Statistical significance was set at *p* < 0.05. Exact *p*-values are reported in the figure legends where possible.

## 3. Results

### 3.1. Candidate Gene Validation

The expression levels of *gspJ*, *gspL*, and *gspM* were assessed by transcriptomic sequencing. Transcriptomic sequencing was previously performed on wild-type *V. alginolyticus* treated with 70 mg/mL *S. baicalensis* extract for 4 h. Based on these data, *gspJ*, *gspL*, and *gspM*, components of the type II secretion system, were identified as significantly downregulated genes. Specifically, after 4 h of treatment, the expression levels of *gspJ*, *gspL*, and *gspM* decreased by 2.28-fold, 1.38-fold, and 1.49-fold, respectively.

To validate these transcriptomic findings, qRT-PCR was performed to independently assess the expression levels of these genes. Wild-type *V. alginolyticus* was treated with 70 mg/mL *S. baicalensis* extract for 4 h, after which total RNA was extracted for analysis.

**Figure 1:**
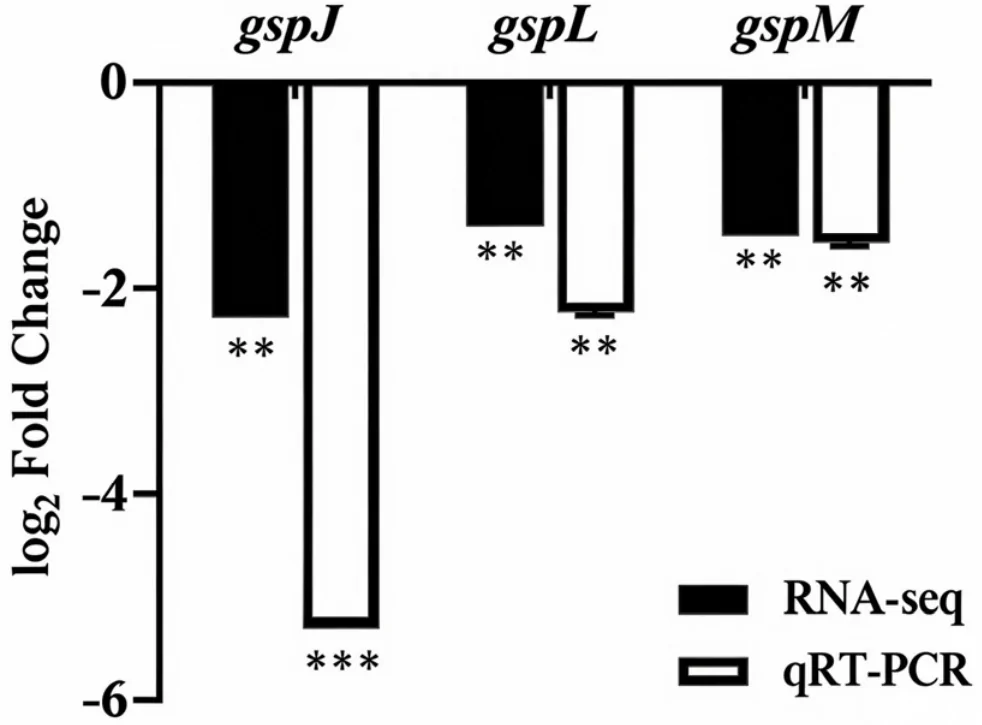
Validation of *gspJ*, *gspL*, and *gspM* expression levels by qRT-PCR. Black bars represent RNA-seq data; white bars represent qRT-PCR results. ** *p* < 0.01; *** *p* < 0.001.

The results showed that the relative expression levels of *gspJ*, *gspL*, and *gspM* were consistent with the transcriptomic data, confirming their reliability and supporting subsequent experiments (Figure 1). We next examined their sequence conservation and predicted protein domains to assess whether their structural features were consistent with conserved T2SS components.

### 3.2. Homologous Sequence Alignment and Domain Prediction for the Purpose of Sequence Characterization

**Figure 2:**
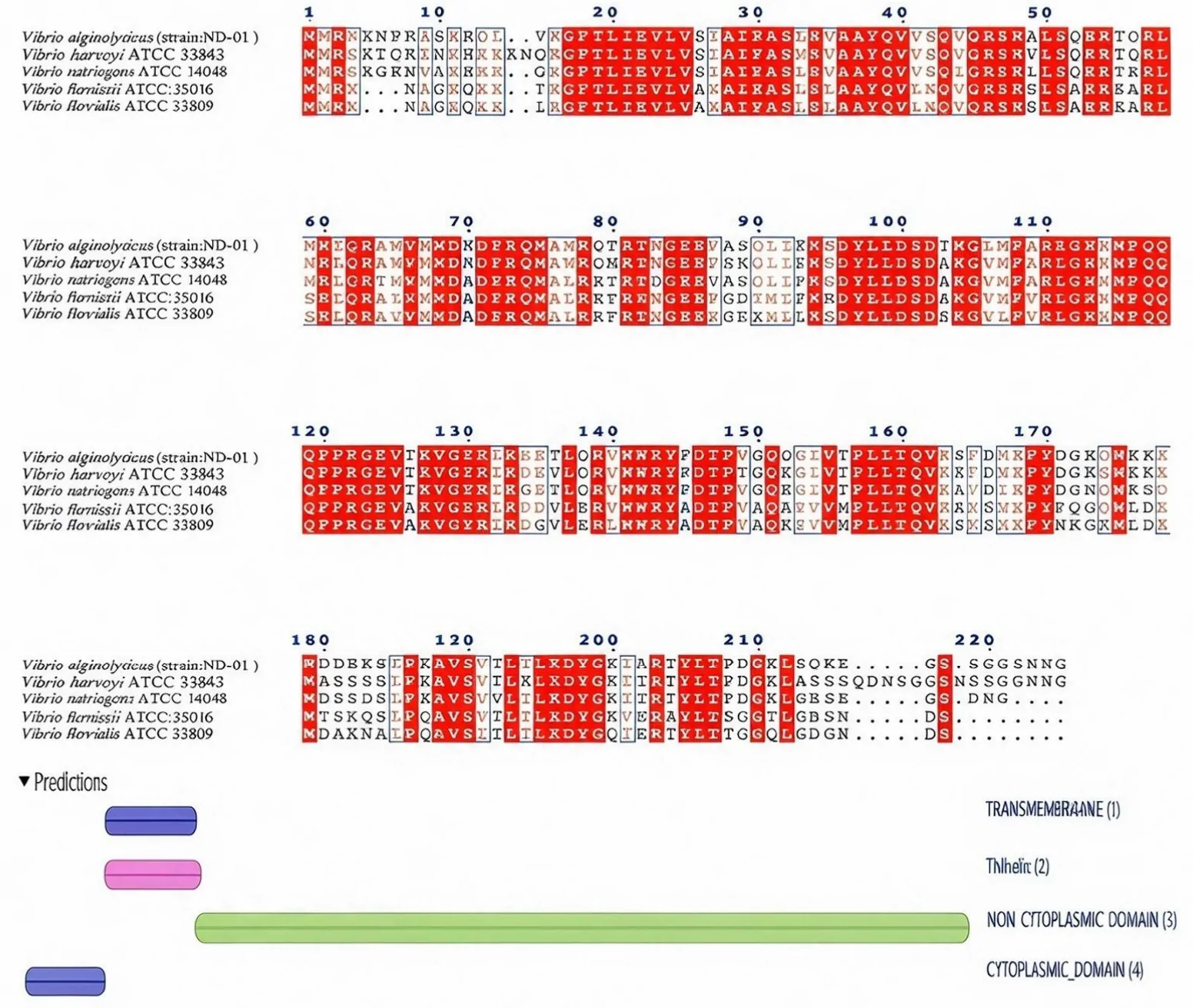
*gspJ* amino acid sequence alignment and domain prediction. Figure note: In the sequence alignment figures, species names are indicated on the left. Amino acid residues highlighted with a red background are fully conserved across all sequences. Residues shown in red text indicate high similarity, while those in black text indicate low similarity or no conservation. The same notation applies to all subsequent alignment figures.

Through homology comparison with functionally characterized genes, the biological function of the target gene can be inferred. Homologous sequences of *gspJ* were retrieved from NCBI, including sequences from *Vibrio harveyi* ATCC 33843, *Vibrio natriegens* ATCC 14048, *Vibrio furnissii* ATCC 5016, and *Vibrio fluvialis* ATCC 33809. As shown in Figure 2, multiple sequence alignment revealed numerous fully conserved residues between positions 20 and 200. Residues at positions 11, 64, 90, 106, 167, and 200 exhibited highly similar physicochemical properties, whereas those at positions 59, 88, 136, 182, and 213 showed weak similarity. These results indicate that *gspJ* is highly conserved within the genus Vibrio and likely shares similar functions with its homologs in related species.

**Figure 3:**
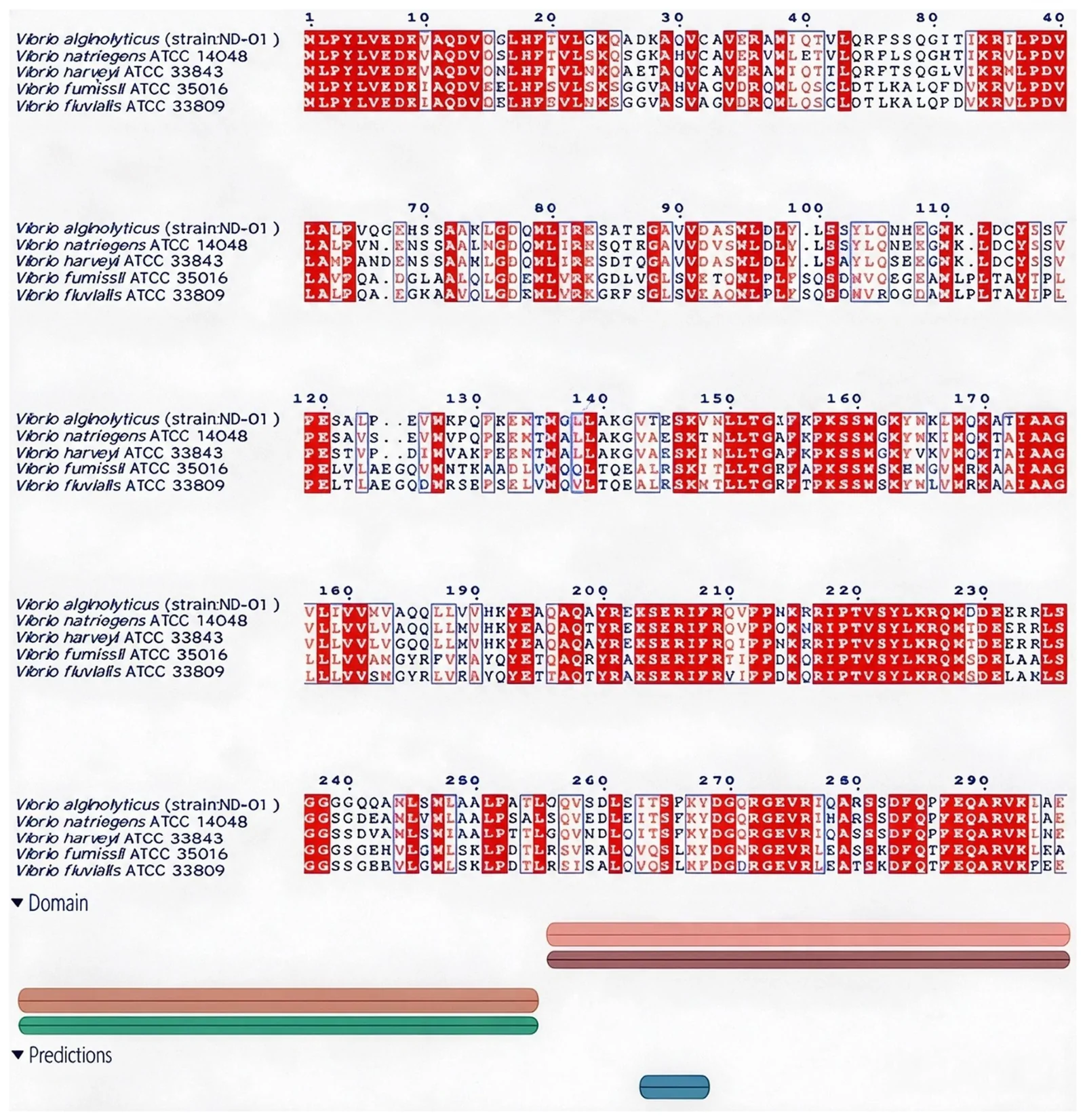
Amino acid sequence alignment and domain prediction of *gspL*. *gspL* amino acid sequence alignment and domain prediction. Figure note: In the sequence alignment figures, species names are indicated on the left. Amino acid residues highlighted with a red background are fully conserved across all sequences. Residues shown in red text indicate high similarity, while those in black text indicate low similarity or no conservation. The same notation applies to all subsequent alignment figures.

Domain prediction using InterPro identified *gspJ* as a membrane-associated protein. As shown in Figure 2, *gspJ* contains a domain spanning residues 20–41, a transmembrane helix spanning residues 20–42, an extracellular region spanning residues 42–225, and a cytoplasmic region spanning residues 1–19. Homologous sequences of *gspL* were also retrieved from NCBI, including sequences from *V. natriegens* ATCC 14048, *V. harveyi* ATCC 33843, *V. furnissii* ATCC 35016, and *V. fluvialis* ATCC 33809. As shown in Figure 3, multiple sequence alignment revealed several fully conserved residues, including those at positions 1, 11, 17, 54, 79, 91, 119, 150, 217, 269, and 287. Residues at positions 39, 68, 77, 103, 133, 171, 187, and 266 exhibited high similarity, whereas those at positions 32, 93, 143, and 239 showed weak similarity. These results suggest that *gspL* is highly conserved within the genus Vibrio and likely shares identical or analogous functions with its homologs in related species.

**Figure 4:**
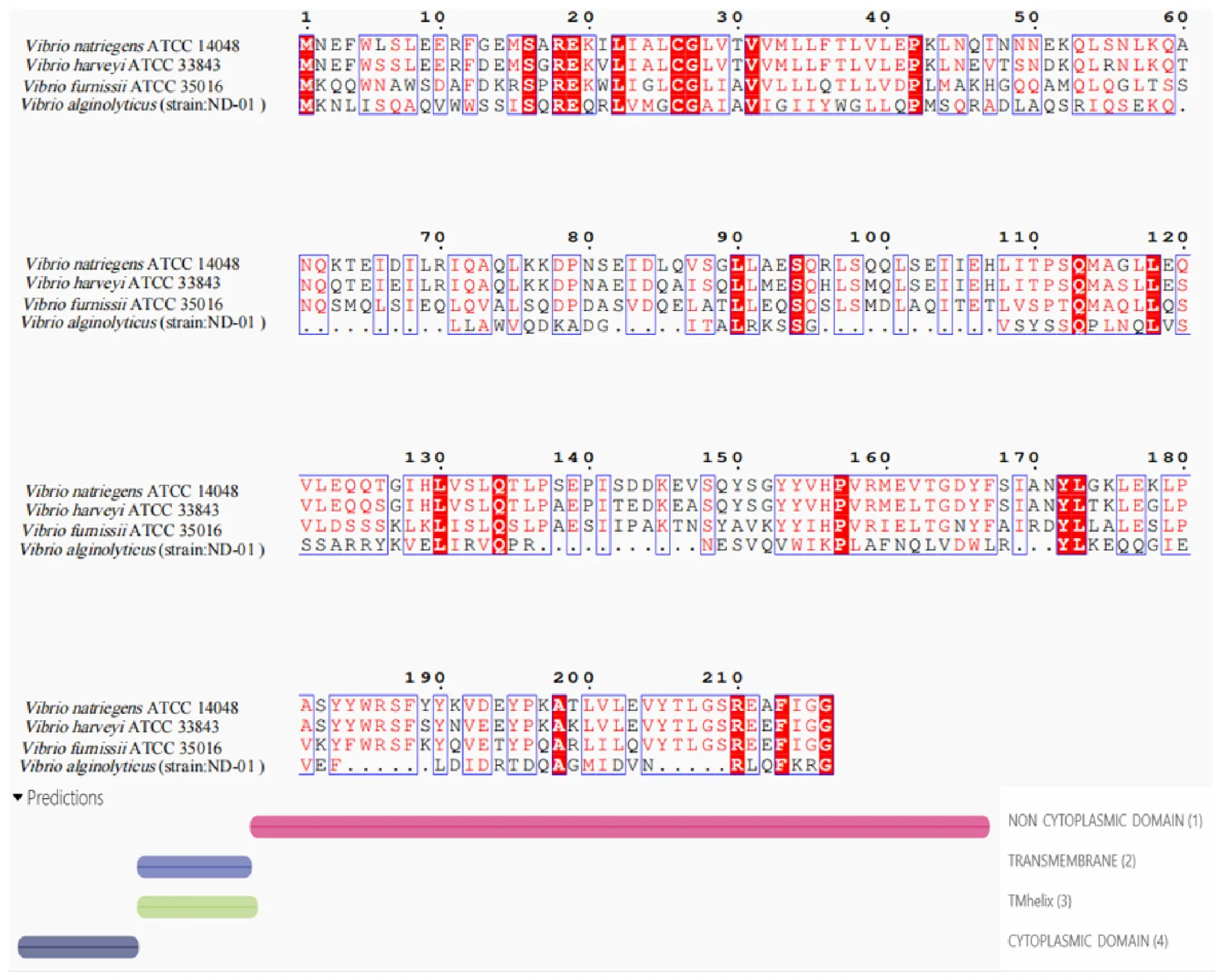
Amino acid sequence alignment and domain prediction of *gspM*. Figure note: In the sequence alignment figures, species names are indicated on the left. Amino acid residues highlighted with a red background are fully conserved across all sequences. Residues shown in red text indicate high similarity, while those in black text indicate low similarity or no conservation. The same notation applies to all subsequent alignment figures.

Domain prediction indicated that *gspL* contains a cytoplasmic region spanning residues 1–158, a periplasmic region spanning residues 162–320, and a coiled-coil region spanning residues 190–210. Homologous sequences of *gspM* were retrieved from NCBI, including sequences from *V. natriegens* ATCC 14048, *V. furnissii* ATCC 35016, and *V. harveyi* ATCC 33843. As shown in Figure 4, alignment revealed fully conserved residues at positions 18, 26, 42, 90, 113, 130, 157, 172, and 198. Residues at positions 2, 12, 29, 39, 75, 87, 98, 128, 154, and 192 exhibited high similarity, whereas those at positions 13, 58, 76, and 181 showed weak similarity. These findings suggest that *gspM* is less conserved than *gspJ* and *gspL* within the genus Vibrio, indicating potential functional divergence. Domain prediction identified *gspM* as a membrane-associated protein, containing an extracellular region (residues 40–163), a transmembrane domain (residues 21–39), a transmembrane helix (residues 21–40), and a cytoplasmic region (residues 1–20). The sequence and domain analyses indicated that all three proteins contained conserved structural features associated with T2SS components. To further characterize their potential structural organization, three-dimensional homology models of GspJ, GspL, and GspM were generated.

### 3.3. Protein Homology Modeling for Structural Modeling

To provide a basis for understanding the biological function and molecular mechanism of GspJ. The amino acid sequence of *gspJ* was used to identify structural templates in SWISS-MODEL. The best-matched template was the EpsJ pseudopilin dimer structure from *Vibrio vulnificus*. The resulting model had a Global Model Quality Estimate (GMQE) value of 0.63, and *gspJ* was predicted to exist as a monomer.

Similarly, the amino acid sequence of *gspL* was used for template identification. The best-matched template was the cytoplasmic domain of the T2SS protein *gspL* from *V. vulnificus*, resolved in complex with the ATPase GspE. The resulting model had a GMQE value of 0.37, and *gspL* was predicted to be monomeric.

**Figure 5:**
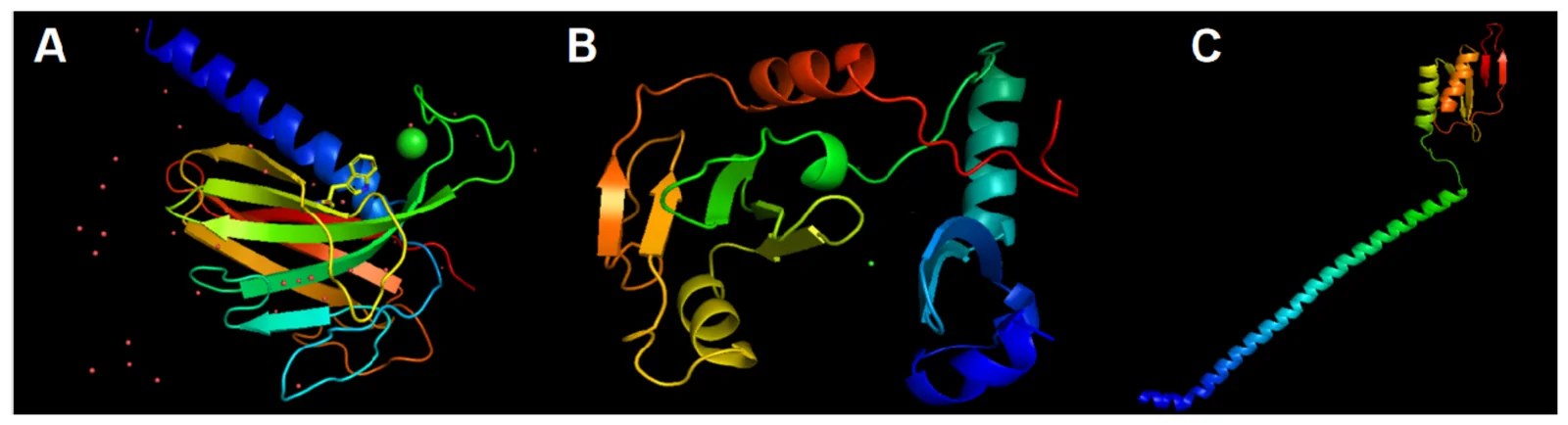
Protein homology modeling of *gspJ*, *gspL*, and *gspM*. (**A**) Three-dimensional homology model of *gspJ*; (**B**) three-dimensional homology model of *gspL*; (**C**) three-dimensional homology model of *gspM*. Cartoon representation of the predicted 3D protein structures. α-helices are shown as spirals, β-strands as arrows indicating the N- to C-terminal direction, and flexible loops as thin lines. The polypeptide chain is colored in rainbow gradient from blue (N-terminus) to red (C-terminus). The green sphere represents bound ligand/metal ion, and red dots represent water molecules (Panel (**A**)).

For *gspM*, the most similar template identified was PilO from the type IV pilus machinery. The resulting model had a GMQE value of 0.57, and *gspM* was also predicted to exist as a monomer (Figure 5). Having characterized the predicted structural features of GspJ, GspL, and GspM, we next investigated their functional contributions by generating stable, gene-silenced strains. Silencing efficiency was first evaluated to identify the strains suitable for subsequent phenotypic analyses.

### 3.4. Validation of Silencing Efficiency

Silencing efficiency in the stable, silenced strains was assessed and validated. Specific shRNAs targeting *gspJ*, *gspL*, and *gspM* were designed and introduced into *V. alginolyticus* via the pACYC184 plasmid through conjugation, generating stably silenced strains. Three shRNAs were designed for each gene, and the construct showing the highest silencing efficiency was used for further experiments.

The resulting strains were as follows: *gspJ*-RNAi-159, *gspJ*-RNAi-315, and *gspJ*-RNAi-562; *gspL*-RNAi-113, *gspL*-RNAi-346, and *gspL*-RNAi-762; and *gspM*-RNAi-148, *gspM*-RNAi-344, and *gspM*-RNAi-441. Silencing efficiency was evaluated by qRT-PCR.

**Figure 6:**
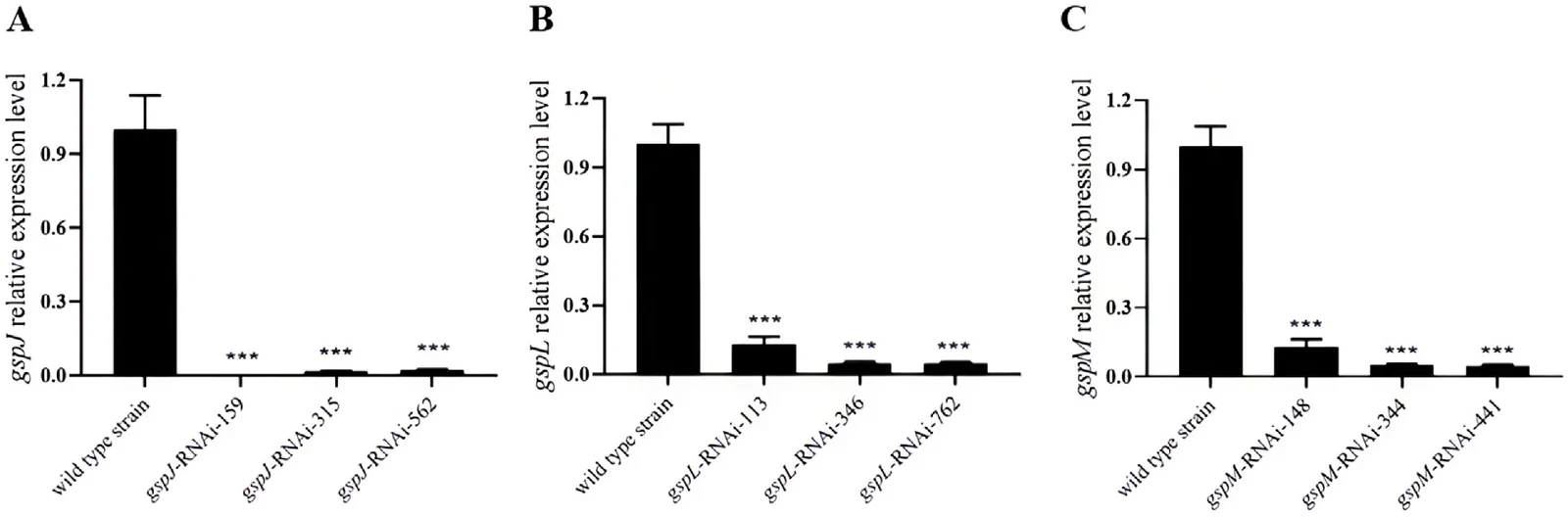
Silencing efficiency of *gspJ*, *gspL*, and *gspM* in stable, silenced strains of *V. alginolyticus*. (**A**) Relative expression of *gspJ* in the *gspJ*-RNAi strain; (**B**) relative expression of *gspL* in the *gspL*-RNAi strain; (**C**) relative expression of *gspM* in the *gspM*-RNAi strain. RNAi, RNA interference. *** *p* < 0.001.

As shown in Figure 6, silencing efficiencies relative to the wild type were 99.57%, 98.27%, and 97.82% for *gspJ* strains; 87.14%, 95.13%, and 95.44% for *gspL* strains; and 89.55%, 94.67%, and 94.62% for *gspM* strains.

The strains that achieved the highest silencing efficiency—*gspJ*-RNAi-159, *gspL*-RNAi-762, and *gspM*-RNAi-344—were selected for all subsequent experiments and are hereafter referred to as *gspJ*-RNAi, *gspL*-RNAi, and *gspM*-RNAi, respectively.

### 3.5. Biological Phenotypes of Silenced Strains

#### 3.5.1. Growth Curve Analysis Results

**Figure 7:**
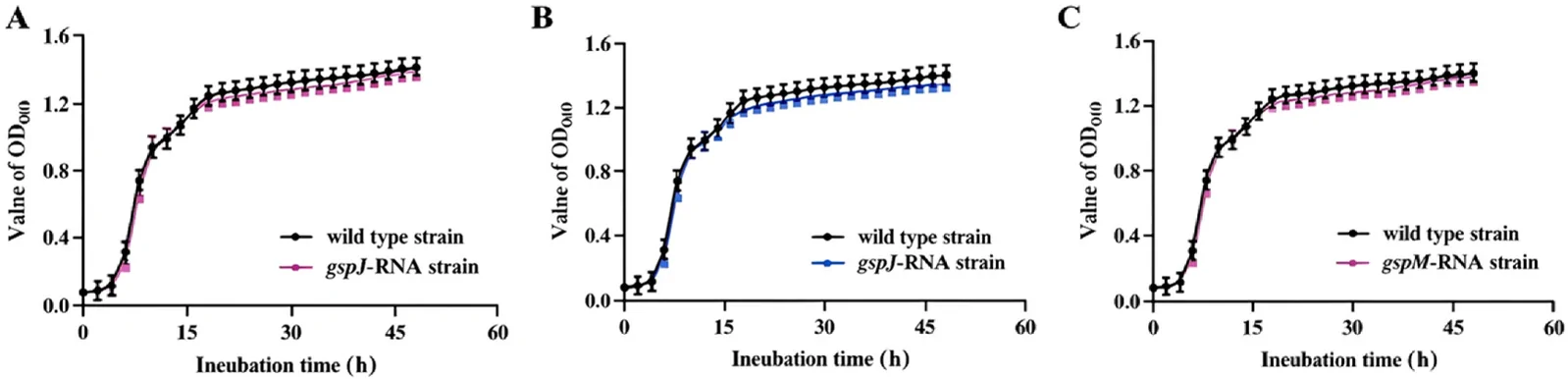
Determination of growth curves of wild strains, *gspJ*-RNAi strain, *gspL*-RNAi strain, and *gspM*-RNAi strain. (**A**) Growth curve determination results of wild strain and *gspJ*-RNAi strain; (**B**) growth curve determination results of wild strain and *gspL*-RNAi strain; (**C**) growth curve determination results of wild strain and *gspM*-RNAi strain.

As shown in Figure 7, the time required for the stable, silenced strains to reach the stationary phase was comparable to that of the wild-type *V. alginolyticus*. Although the total biomass of the silenced strains in the stationary phase was marginally lower than the corresponding value for wild-type *V. alginolyticus*, the general growth trends were similar. The results demonstrate that silencing of the target genes has no effect on bacterial growth, confirming that the silenced strains are suitable for subsequent experiments.

#### 3.5.2. Biofilm Formation Assay Results

**Figure 8:**
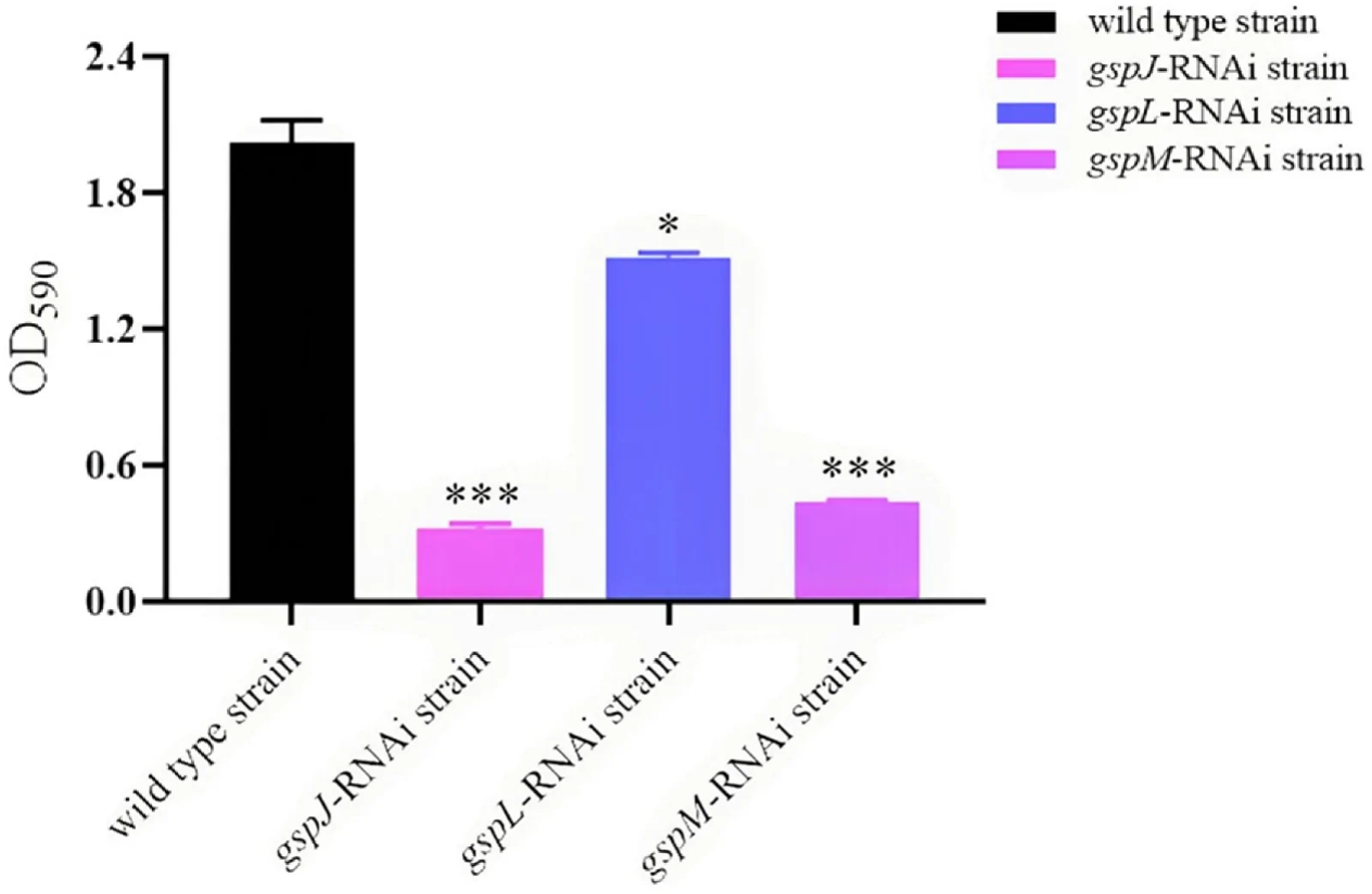
Determination of biofilm formation ability of wild-type *V. alginolyticus*, *gspJ*-RNAi strain, *gspL*-RNAi strain, and *gspM*-RNAi strain. * *p* < 0.05, *** *p* < 0.001.

As shown in Figure 8, in comparison with the wild-type *V. alginolyticus*, biofilm formation ability was reduced by 84.01% in the *gspJ*-RNAi strain, 24.99% in the *gspL*-RNAi strain, and 78.26% in the *gspM*-RNAi strain. The marked changes in biofilm formation prompted further assessment of other traits associated with bacterial colonization, including motility, adhesion, and chemotaxis.

#### 3.5.3. Motility Assay Results

**Figure 9:**
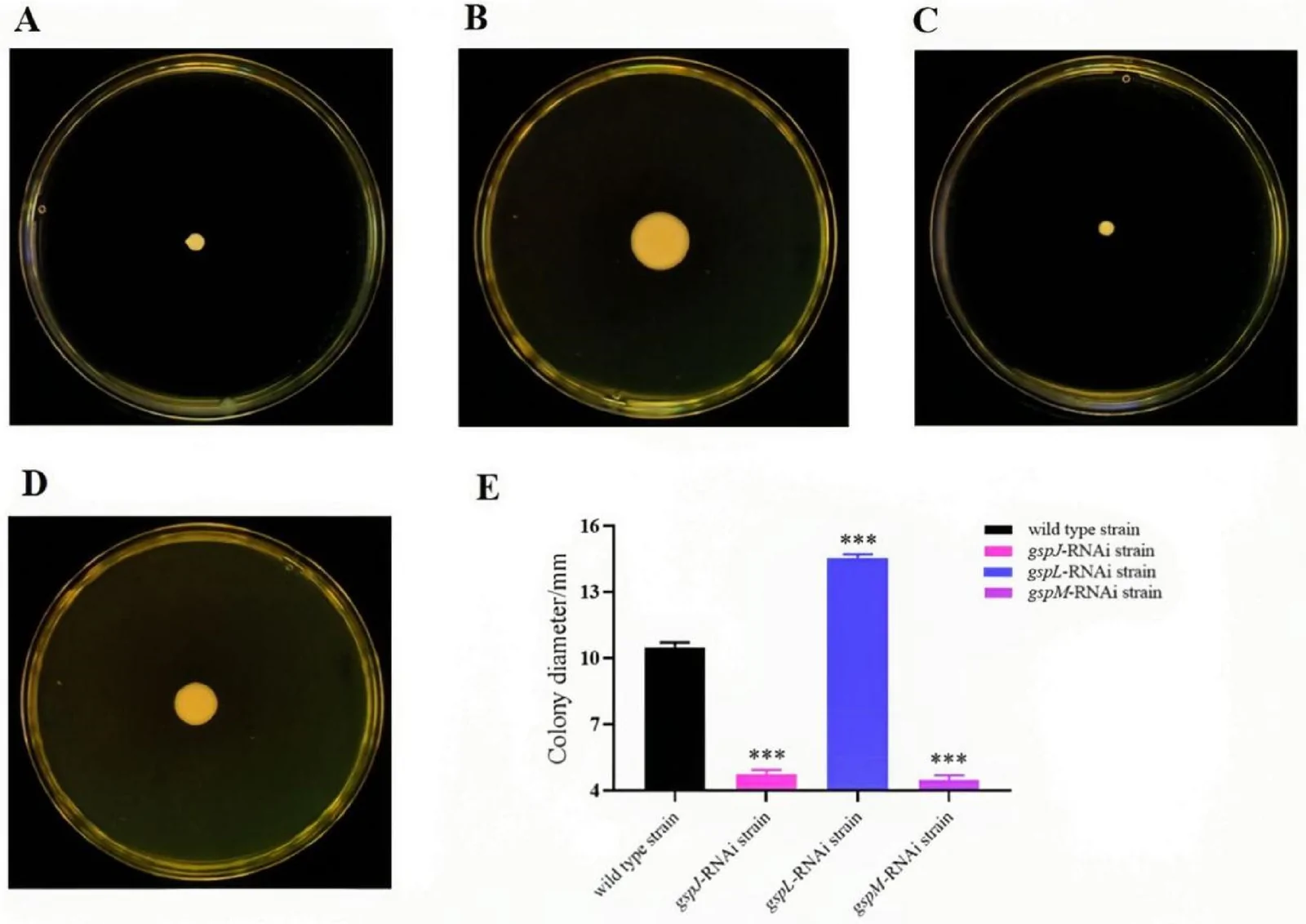
Motility test results of wild strains, *gspJ*-RNAi strain, *gspL*-RNAi strain, and *gspM*-RNAi strain. (**A**) Colony formation of stable, silent strain *gspJ*-RNAi on semi-solid culture medium; (**B**) colony formation of stable, silent strain *gspL*-RNAi on semi-solid culture medium; (**C**) colony formation of stable, silent strain *gspM*-RNAi on semi-solid culture medium; (**D**) colony formation of the wild-type strain on semi-solid culture medium; (**E**) colony diameter bar chart. *** *p* < 0.001.

As shown in Figure 9, motility was reduced by 54.82% in the *gspJ*-RNAi strain and 57.42% in the *gspM*-RNAi strain, whereas it increased by 38.49% in the *gspL*-RNAi strain in comparison with the wild-type *V. alginolyticus*.

#### 3.5.4. Bacterial Adhesion Assay Results

**Figure 10:**
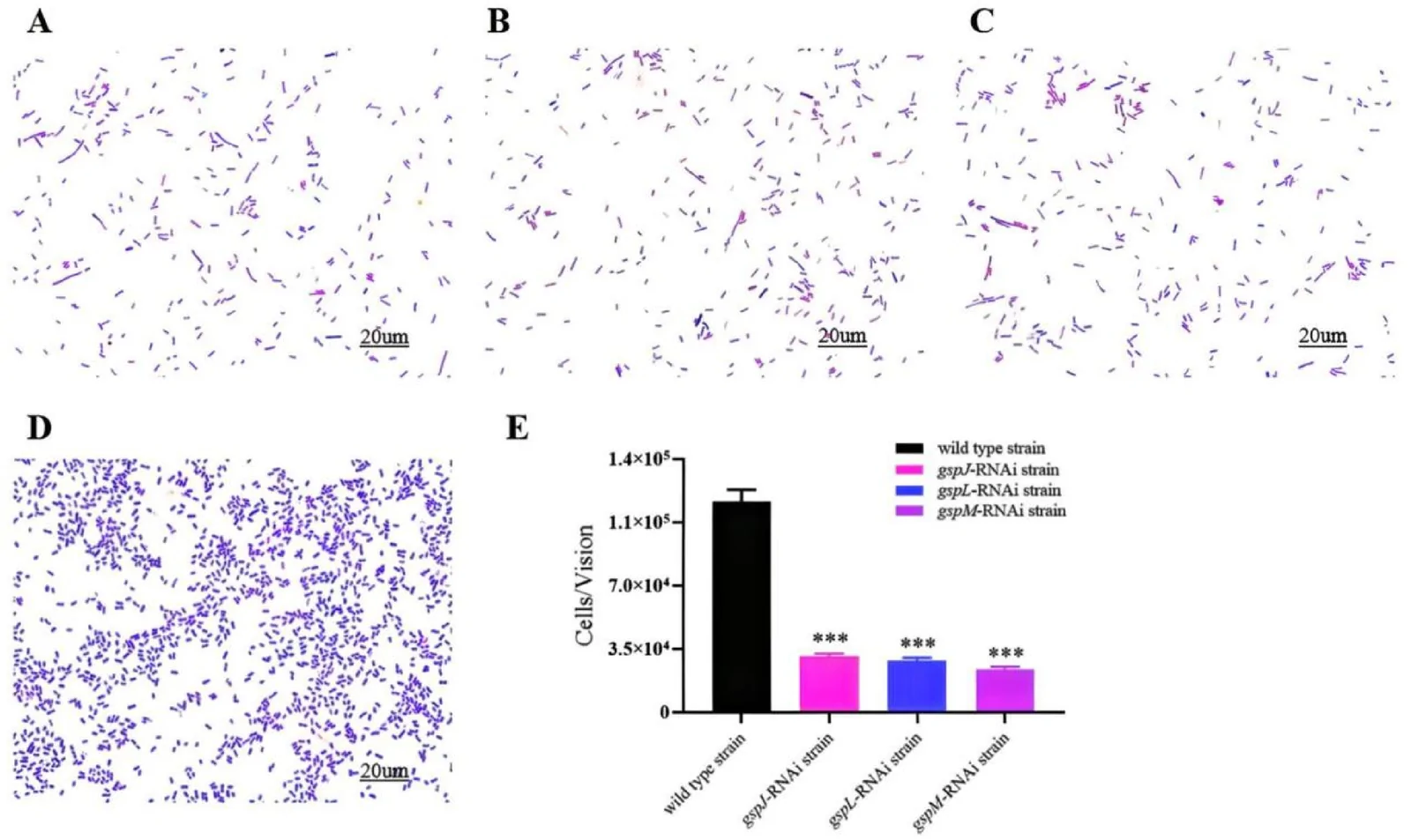
Measurement results of adhesion ability of wild strains, *gspJ*-RNAi strain, *gspL*-RNAi strain, and *gspM*-RNAi strain. (**A**) Adhesion ability test results of silent strain *gspJ*-RNAi; (**B**) adhesion ability test results of silent strain *gspL*-RNAi; (**C**) adhesion ability test results of silent strain *gspM*-RNAi; (**D**) adhesion ability test results of the wild-type strain; (**E**) statistical histogram of adhesion data. *** *p* < 0.001.

As shown in Figure 10, the number of adherent bacterial cells observed under microscopy was significantly reduced in all three silenced strains compared with the wild-type *V. alginolyticus*. Adhesion decreased by 73.57% in the *gspJ*-RNAi strain, 75.65% in the *gspL*-RNAi strain, and 79.61% in the *gspM*-RNAi strain.

#### 3.5.5. Bacterial Chemotaxis Assay Results

**Figure 11:**
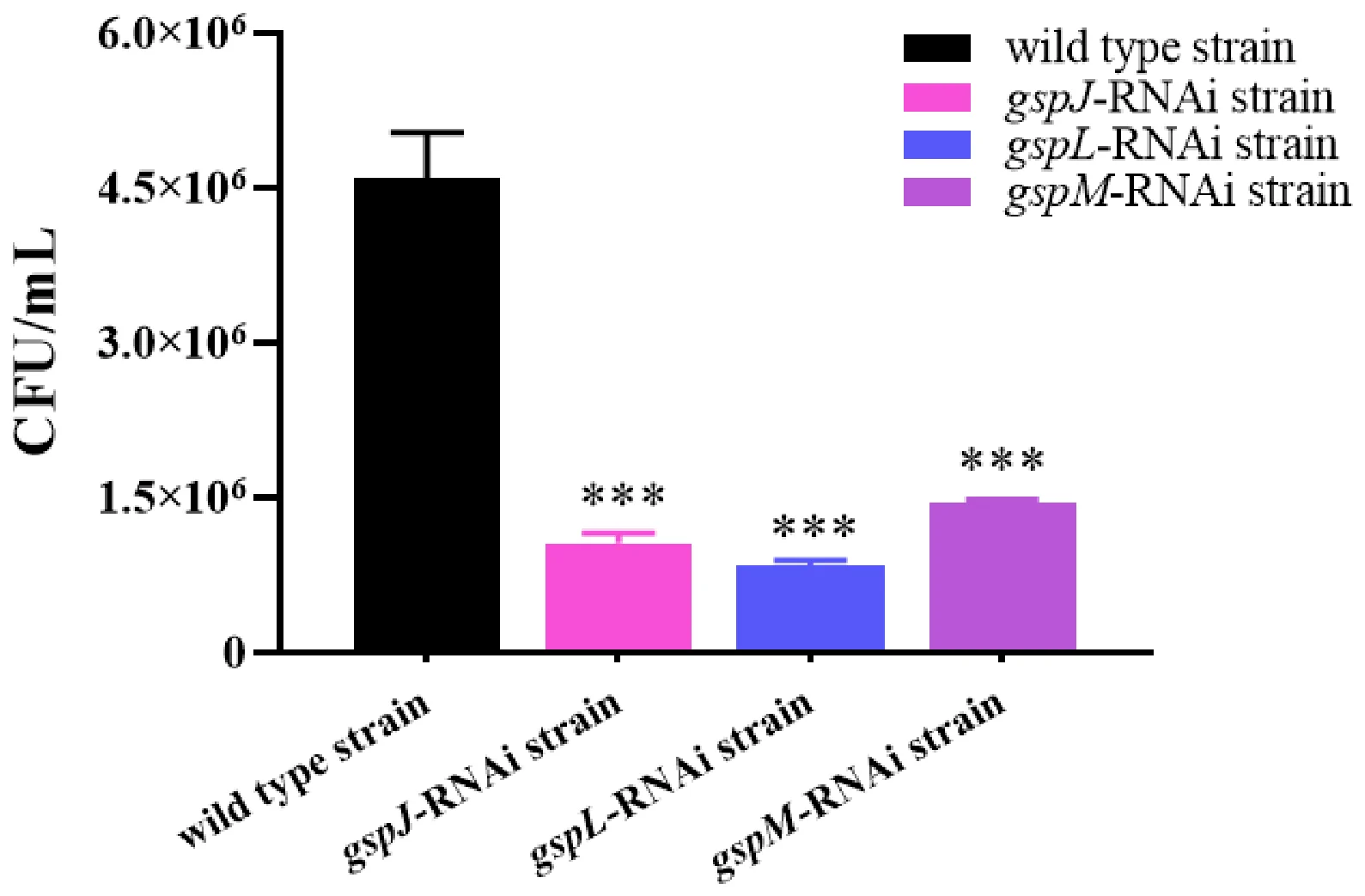
Determination of chemotaxis of wild-type strains and *gspJ*-RNAi, *gspL*-RNAi, and *gspM*-RNAi strains. *** *p* < 0.001.

As shown in Figure 11, chemotaxis was significantly reduced in all three silenced strains in comparison with the wild-type *V. alginolyticus*. Chemotaxis decreased by 76.87% in the *gspJ*-RNAi strain, 81.80% in the *gspL*-RNAi strain, and 68.38% in the *gspM*-RNAi strain.

### 3.6. Environmental Regulation of gspJ, gspL, and gspM Expression

#### 3.6.1. Responses to pH Variation

**Figure 12:**
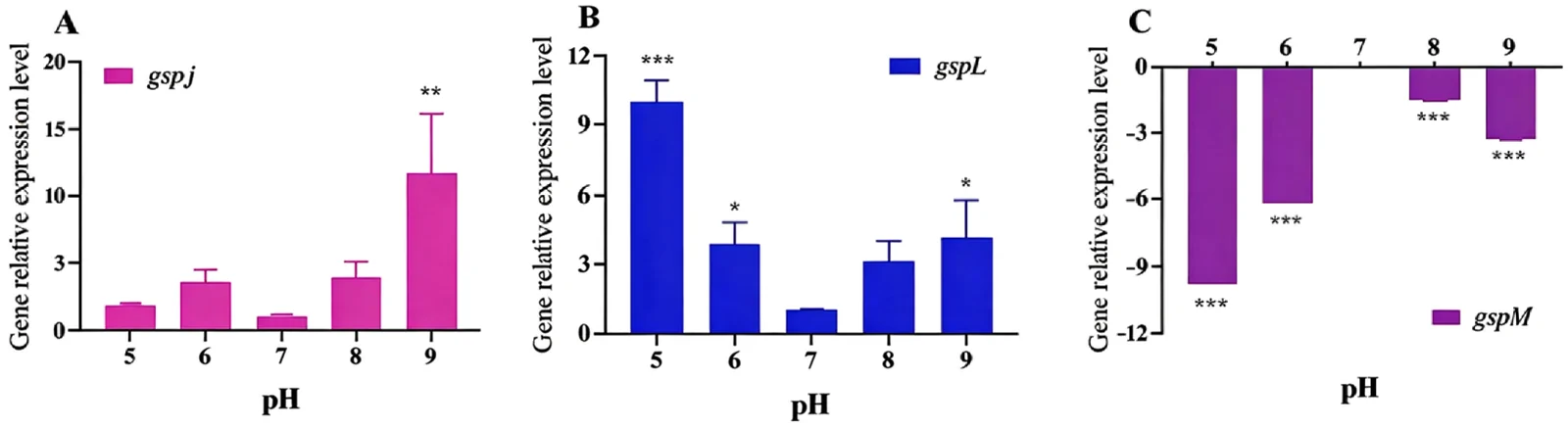
Expression levels of *gspJ*, *gspL*, and *gspM* under different pH conditions. (**A**) Expression levels of *gspJ* under different pH conditions; (**B**) expression levels of *gspL* under different pH values; (**C**) expression levels of *gspM* under different pH values; the pH 7 group was used as the control, with its expression level normalized to 1. * *p* < 0.05, ** *p* < 0.01, *** *p* < 0.001.

After establishing the effects of gene silencing on virulence-associated phenotypes, we next examined whether *gspJ*, *gspL*, and *gspM* transcription was responsive to environmental variation. Because pH and temperature are major environmental variables in aquatic systems, their effects were evaluated separately. Wild-type *V. alginolyticus* was treated with pH stress, and the expression levels of *gspJ*, *gspL*, and *gspM* under five pH conditions are shown in Figure 12.

Using pH 7, the optimal growth pH, as the control, the expression of *gspJ* increased by 0.82-fold, 2.63-fold, 3.00-fold, and 10.69-fold at pH 5, 6, 8, and 9, respectively. The expression of *gspL* increased by 9.03-fold, 2.87-fold, 2.11-fold, and 3.14-fold at pH 5, 6, 8, and 9, respectively.

In contrast, the expression of *gspM* decreased by 99.89%, 98.61%, 65.08%, and 89.66% at pH 5, 6, 8, and 9, respectively.

#### 3.6.2. Responses to Temperature Variation

**Figure 13:**
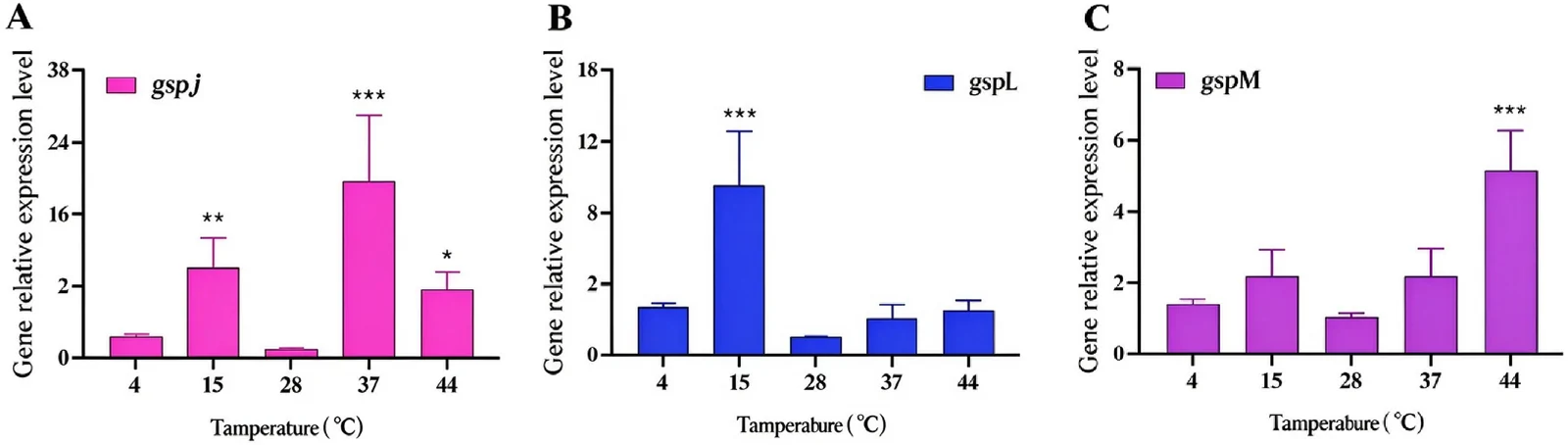
Expression levels of *gspJ*, *gspL*, and *gspM* under different temperature conditions. (**A**) Expression levels of *gspJ* under different temperature conditions; (**B**) expression levels of *gspL* under different temperature conditions; (**C**) expression levels of *gspM* under different temperature conditions; the 28 °C-treatment group was used as the control, with its expression level normalized to 1. * *p* < 0.05, ** *p* < 0.01, *** *p* < 0.001.

Wild-type *V. alginolyticus* was subjected to temperature stress, and the expression levels of *gspJ*, *gspL*, and *gspM* under different temperature conditions are shown in Figure 13.

Using 28 °C, the optimal growth temperature, as the control, the expression of *gspJ* exhibited an increase of 1.42-fold, 9.11-fold, 18.77-fold, and 6.73-fold at 4 °C, 15 °C, 37 °C, and 44 °C, respectively. The expression of *gspL* exhibited an increase of 1.69-fold, 8.56-fold, 1.09-fold, and 1.55-fold at 4 °C, 15 °C, 37 °C, and 44 °C, respectively.

Similarly, the expression of *gspM* exhibited an increase of 0.41-fold, 1.18-fold, 1.18-fold, and 4.13-fold at 4 °C, 15 °C, 37 °C, and 44 °C, respectively.

### 3.7. In Vivo Virulence of the gspL-RNAi Strain

#### 3.7.1. Artificial Infection

Hybrid groupers were infected with either wild-type *V. alginolyticus* or the *gspL*-RNAi strain, and survival was measured for a 14-day period.

**Figure 14:**
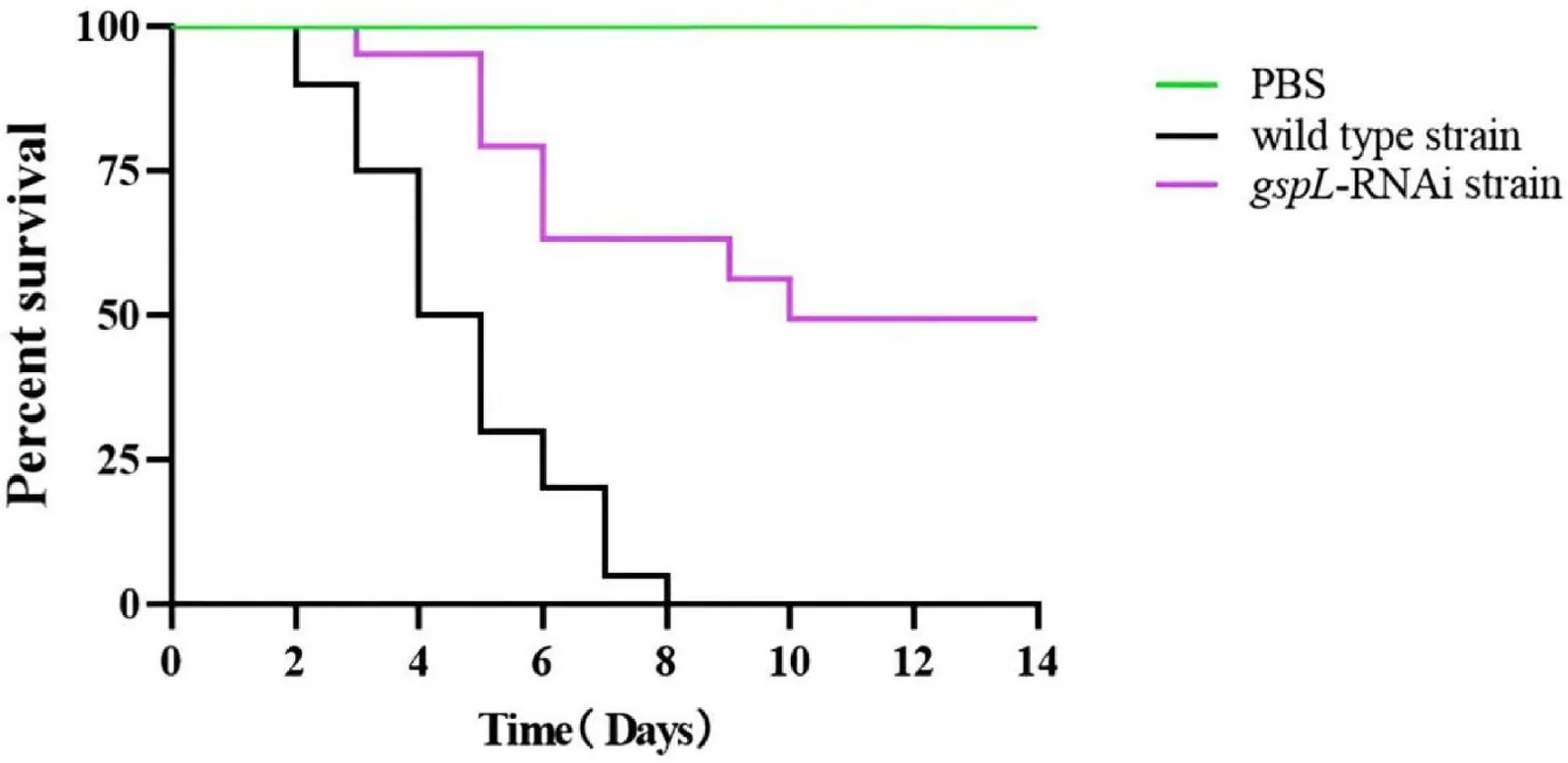
Survival of *E. coioides* exposed to wild-type *V. alginolyticus* and *gspL*-RNAi strain. The phosphate-buffered saline (PBS) group served as the negative control. RNAi, RNA interference; PBS, phosphate-buffered saline.

As shown in Figure 14, the survival rate of the PBS control group remained at 100% throughout the experiment. In the wild-type *V. alginolyticus* infection group, mortality started on day 2 post-infection, and the survival rate decreased to 0% by day 8.

In contrast, in the *gspL*-RNAi infection group, mortality onset was delayed to day 3, and cumulative mortality was reduced by 50% in comparison with the wild-type *V. alginolyticus* group.

#### 3.7.2. Bacterial Load

**Figure 15:**
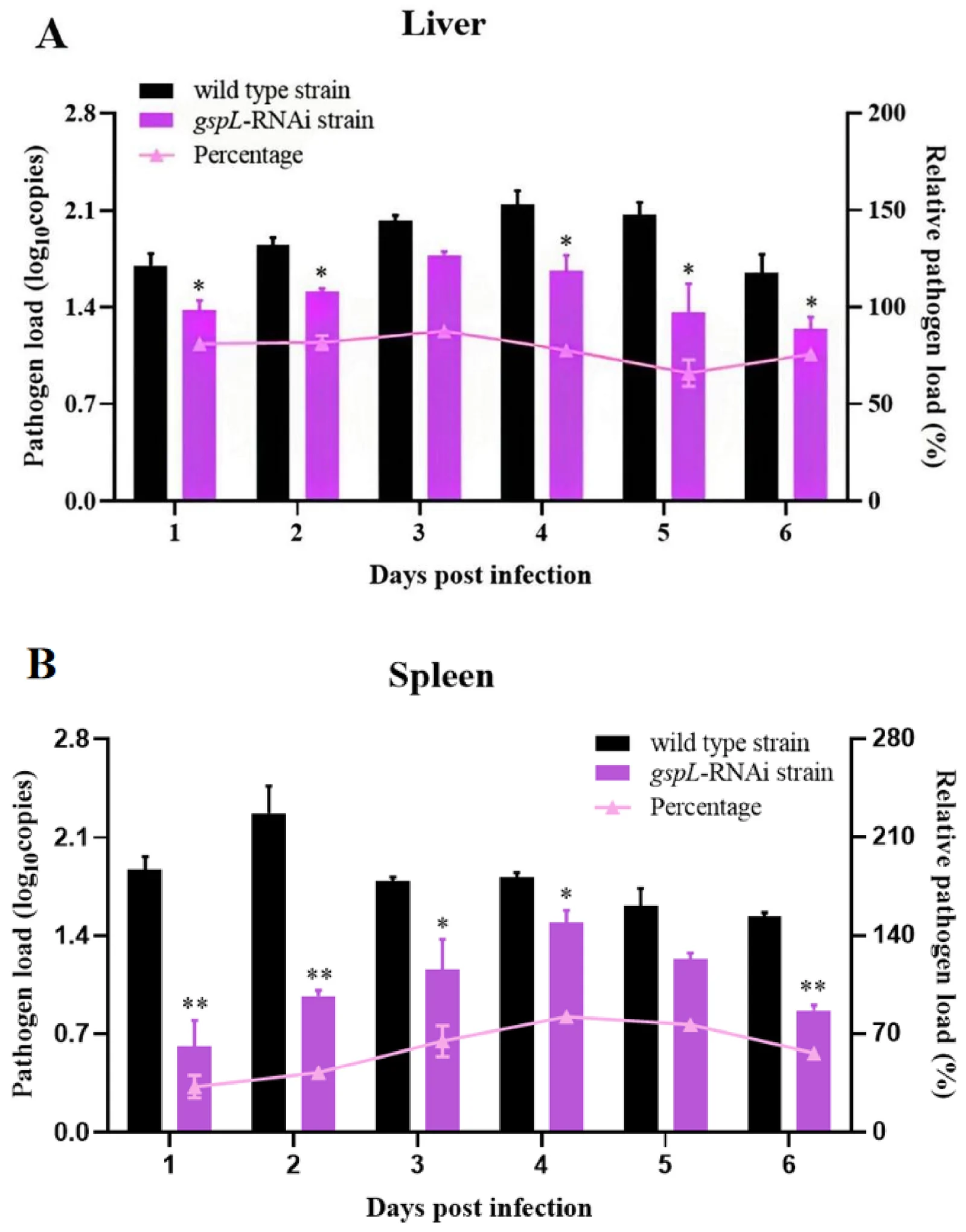
Bacterial load in *E. coioides* challenged with wild-type *V. alginolyticus* and *gspL*-RNAi strain. (**A**) Liver bacterial load in *E. coioides* following artificial challenge; (**B**) spleen bacterial load in *E. coioides* following artificial challenge. * *p* < 0.05, ** *p* < 0.01.

The survival analysis indicated attenuated pathogenicity of the *gspL*-RNAi strain. To determine whether this attenuation was accompanied by reduced tissue colonization, bacterial loads were subsequently quantified in the liver and spleen. As shown in Figure 15, bacterial loads of both wild-type *V. alginolyticus* and the *gspL*-RNAi strain in the liver followed a pattern of initial increase followed by decline, peaking on day 4 and day 3, respectively. Throughout the infection period, the bacterial load in the *gspL*-RNAi strain stayed consistently lower than that in the wild-type *V. alginolyticus*.

In the spleen, bacterial loads of the wild-type *V. alginolyticus* and *gspL*-RNAi strain peaked on day 2 and day 4, respectively. The load of the *gspL*-RNAi strain remained consistently lower than that of the wild-type *V. alginolyticus* throughout the infection period.

Given the attenuated in vivo pathogenicity of the *gspL*-RNAi strain, we next used label-free proteomics to investigate the broader molecular changes associated with gspL silencing.

### 3.8. Label-Free Proteomic Analysis Results

#### 3.8.1. Identification Statistics

The identification results were as follows: total spectra, 638,459; matched spectra, 63,613; total peptides, 16,515; unique peptides, 15,164; identified proteins, 2303; and quantified proteins, 2158.

#### 3.8.2. Differential Expression Analysis

Differentially expressed proteins were identified using the following criteria: fold change (FC) > 2 (upregulated) or <0.5 (downregulated) and *p* < 0.05 [12,13]. A total of 189 differentially expressed proteins were identified, including 45 upregulated and 144 downregulated proteins. Among these, three proteins were upregulated more than 10-fold, and six proteins were downregulated more than 10-fold.

**Figure 16:**
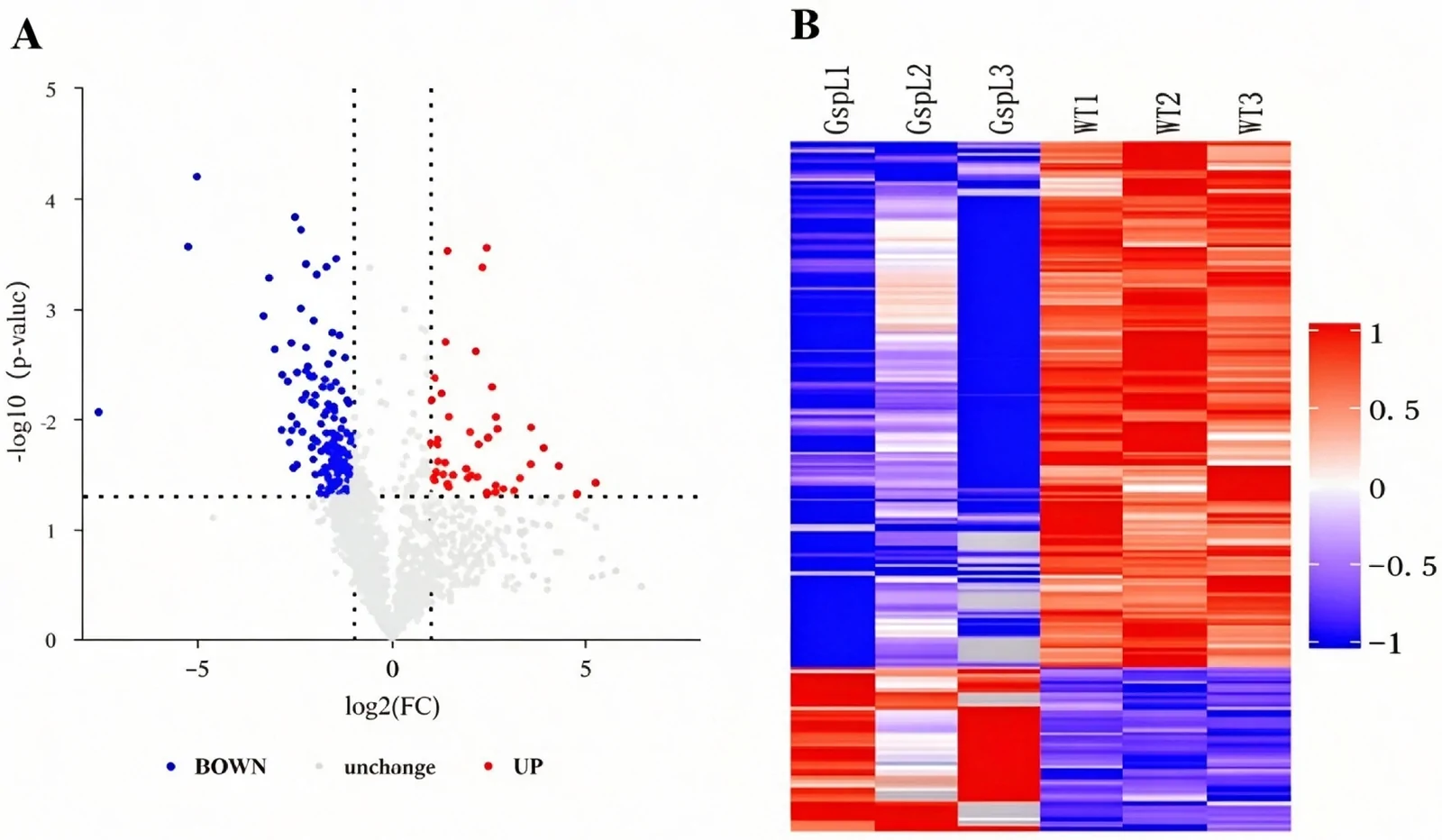
Differential protein expression between the wild-type and *gspL*-RNAi strains. (**A**) Volcano plot of differentially expressed proteins; (**B**) hierarchical clustering heatmap of differentially expressed proteins. RNAi, RNA interference; FC, fold change.

Differential protein expression between groups was visualized using a volcano plot based on fold change and *p*-value (Figure 16A). To assess sample clustering consistency, hierarchical clustering analysis was performed and presented as a heatmap (Figure 16B). The clear separation of groups indicates that the identified proteins reliably reflect the biological differences between treatment and control samples.

#### 3.8.3. Subcellular Localization

Subcellular organelles are functional microstructures within the cytoplasm (e.g., mitochondria and the endoplasmic reticulum) that serve as key sites for diverse protein functions [13]. As different organelles perform distinct cellular roles, analysis of protein subcellular localization provides insight into their potential functions [12].

**Figure 17:**
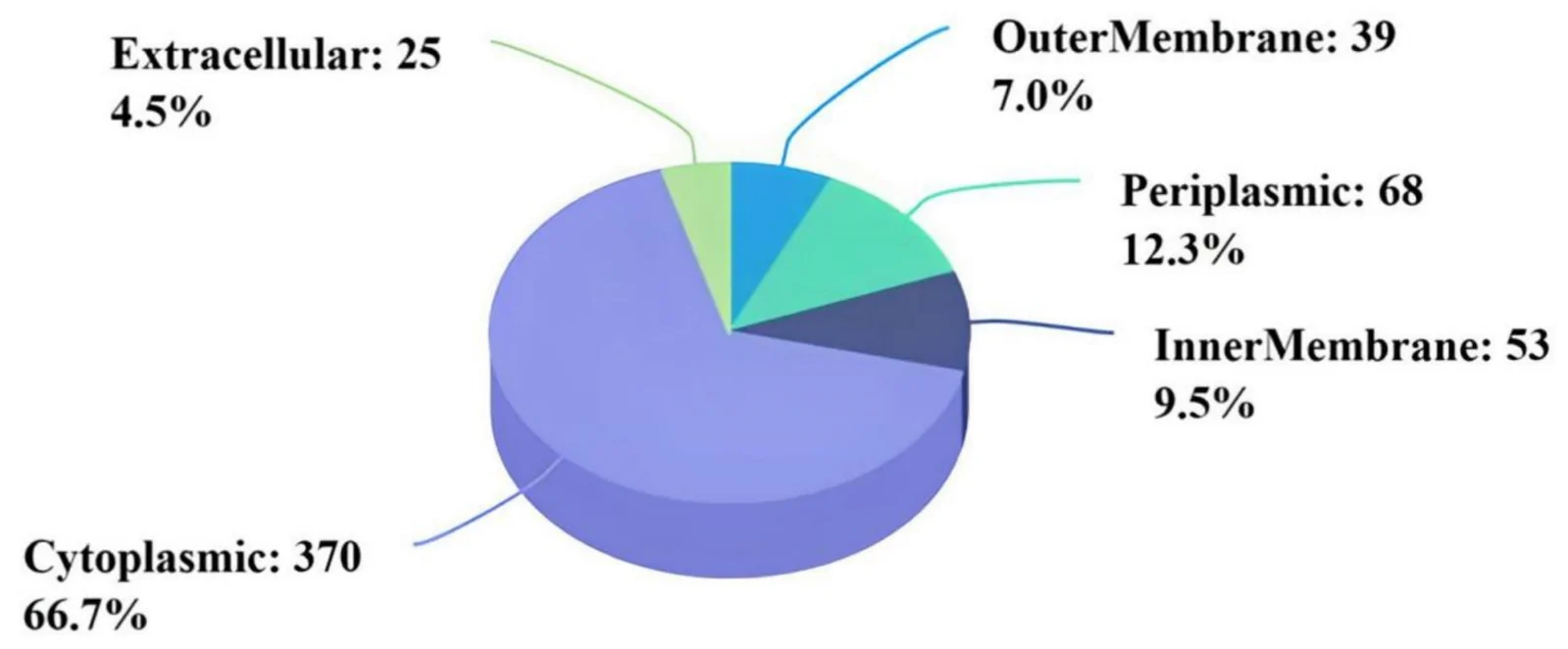
Subcellular localization of differentially expressed proteins. Cytoplasm: 370 proteins (66.7%); periplasm: 68 proteins (12.3%); inner membrane: 53 proteins (9.5%); outer membrane: 39 proteins (7.0%); extracellular: 25 proteins (4.5%).

All differentially expressed proteins were subjected to subcellular localization prediction using CELLO v.2.5 software, with 555 proteins successfully assigned. As shown in Figure 17, the majority of differentially expressed proteins were localized to the cytoplasm, accounting for 66.7% of the total. The remaining proteins were mainly distributed in the periplasmic space, inner membrane, and outer membrane, whereas the smallest proportion (4.5%) was localized extracellularly.

#### 3.8.4. Domain Analysis

**Figure 18:**
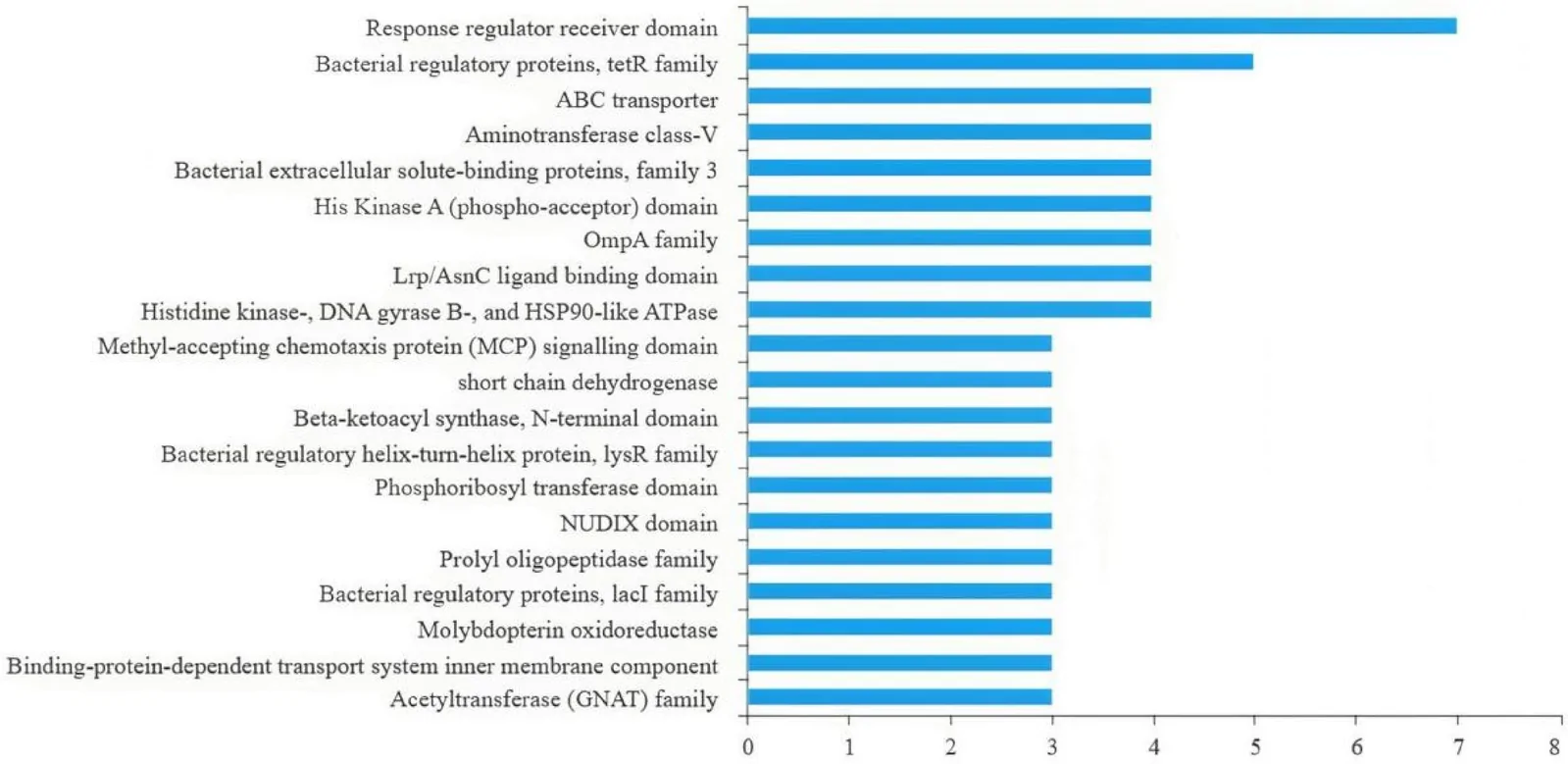
Prediction of differential protein domains. The horizontal axis represents the number of differential proteins enriched in different structural domains, while the vertical axis represents the domain name.

Domain prediction of the differentially expressed proteins was performed using InterProScan. The number of proteins associated with the top 20 enriched domains is shown in Figure 18. The differentially expressed proteins were primarily enriched in the following domains: response regulator receiver domain, TetR family transcriptional regulator, ABC transporter system, class V aminotransferase, bacterial extracellular solute-binding protein, His kinase A (phosphoacceptor) domain, OmpA family, and Lrp/AsnC ligand-binding domain. Among these, the response regulator receiver domain had the highest number of associated proteins (7), followed by the TetR family (5). The ABC transporter system, class V aminotransferase, extracellular solute-binding proteins, His kinase A domain, OmpA family, and Lrp/AsnC domain each included four proteins. Other domains, including the methyl-accepting chemotaxis protein signaling domain, phosphoribosyl transferase domain, bacterial regulatory protein domain, and prolyl oligopeptidase domain, each included three proteins.

#### 3.8.5. GO Functional Analysis

**Figure 19:**
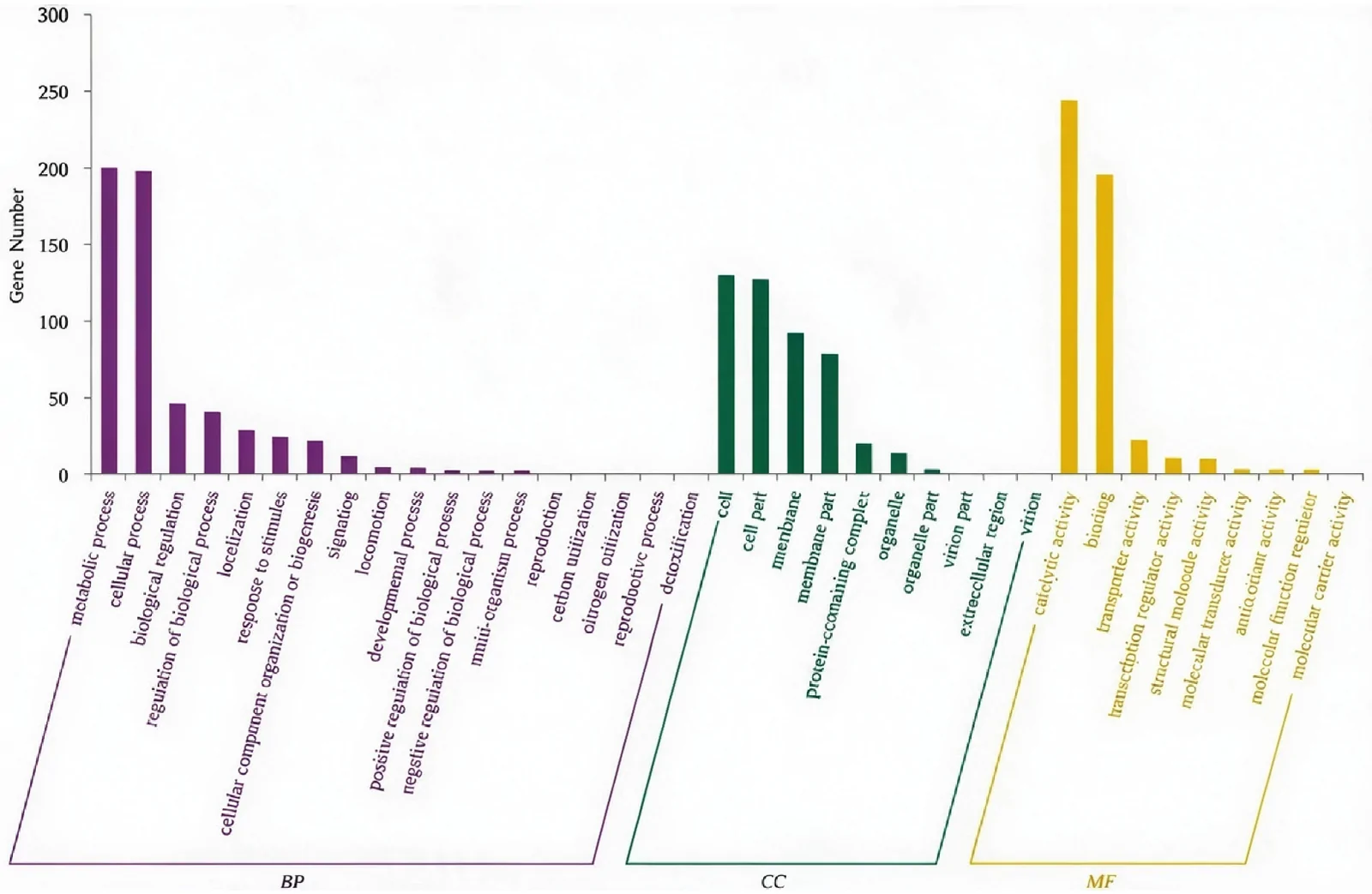
Gene Ontology (GO) enrichment map to classify differentially expressed proteins. GO, Gene Ontology.

GO functional annotation of all differentially expressed proteins was performed using Blast2GO (https://www.blast2go.com/ accessed on 25 August 2026), and the number of proteins in each secondary GO category was determined. As shown in Figure 19, within the Biological Process category, the differentially expressed proteins were primarily enriched in metabolic and cellular processes. Within the Cellular Component category, enrichment was mainly observed in the cell, membrane, and membrane part categories. Within the Molecular Function category, proteins were predominantly enriched in catalytic activity and binding.

**Figure 20:**
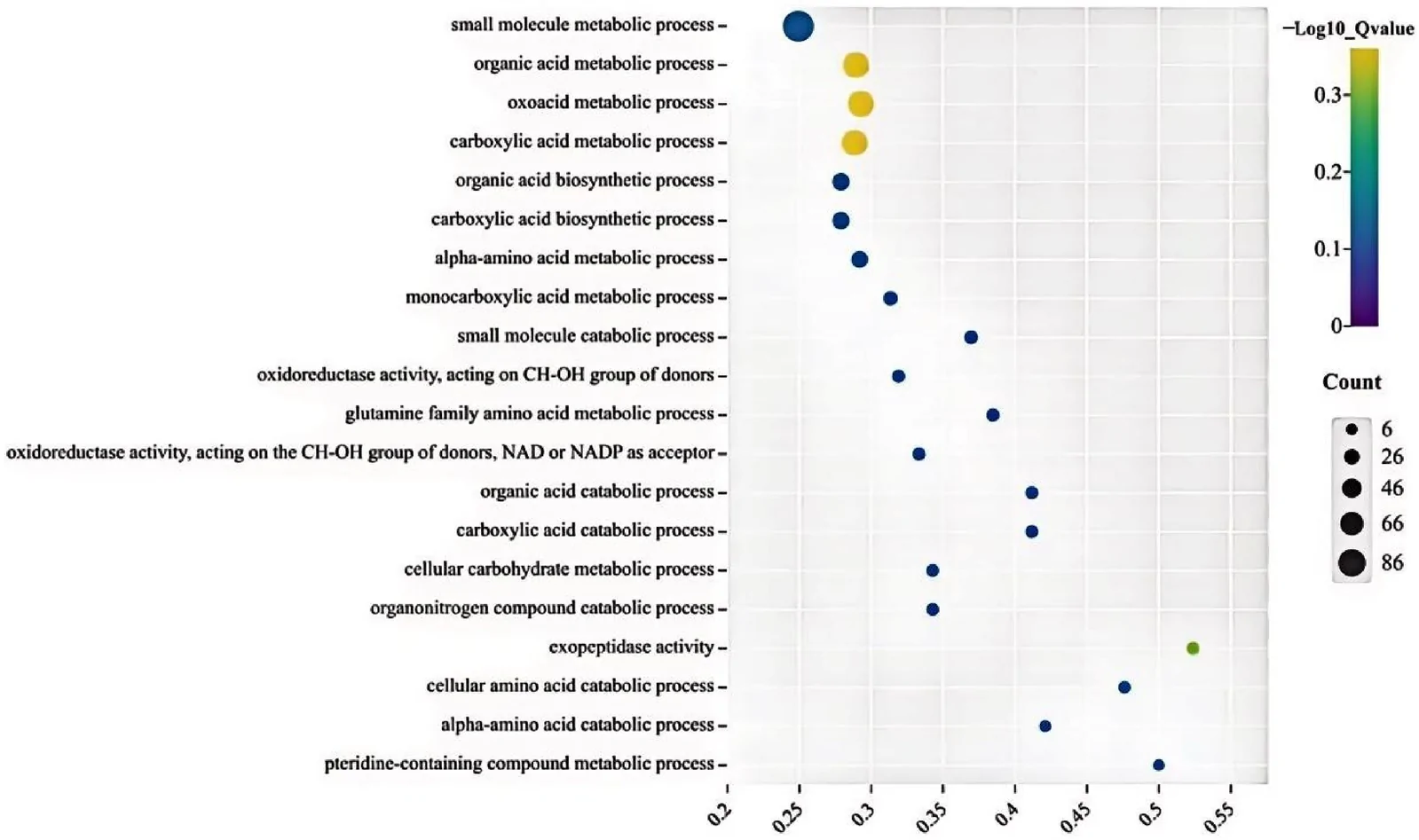
GO scatter chart to classify differentially expressed proteins.

To further characterize functional enrichment, GO enrichment analysis was performed using Fisher’s exact test (*p* < 0.05) [17]. As shown in Figure 20, the differentially expressed proteins were mainly enriched in biological processes related to small molecule metabolism, including organic acid, oxoacid, carboxylic acid, and amino acid metabolic and biosynthetic processes, as well as small molecule catabolic processes.

Among these, the largest number of proteins (105) were enriched in the small molecule metabolic process, followed by the organic acid metabolic process (75 proteins), oxoacid metabolic process (75 proteins), and carboxylic acid metabolic process (73 proteins). The enrichment significance of organic acid-, oxoacid-, and carboxylic acid-related processes was notably higher than that of other categories.

#### 3.8.6. KEGG Pathway Analysis

**Figure 21:**
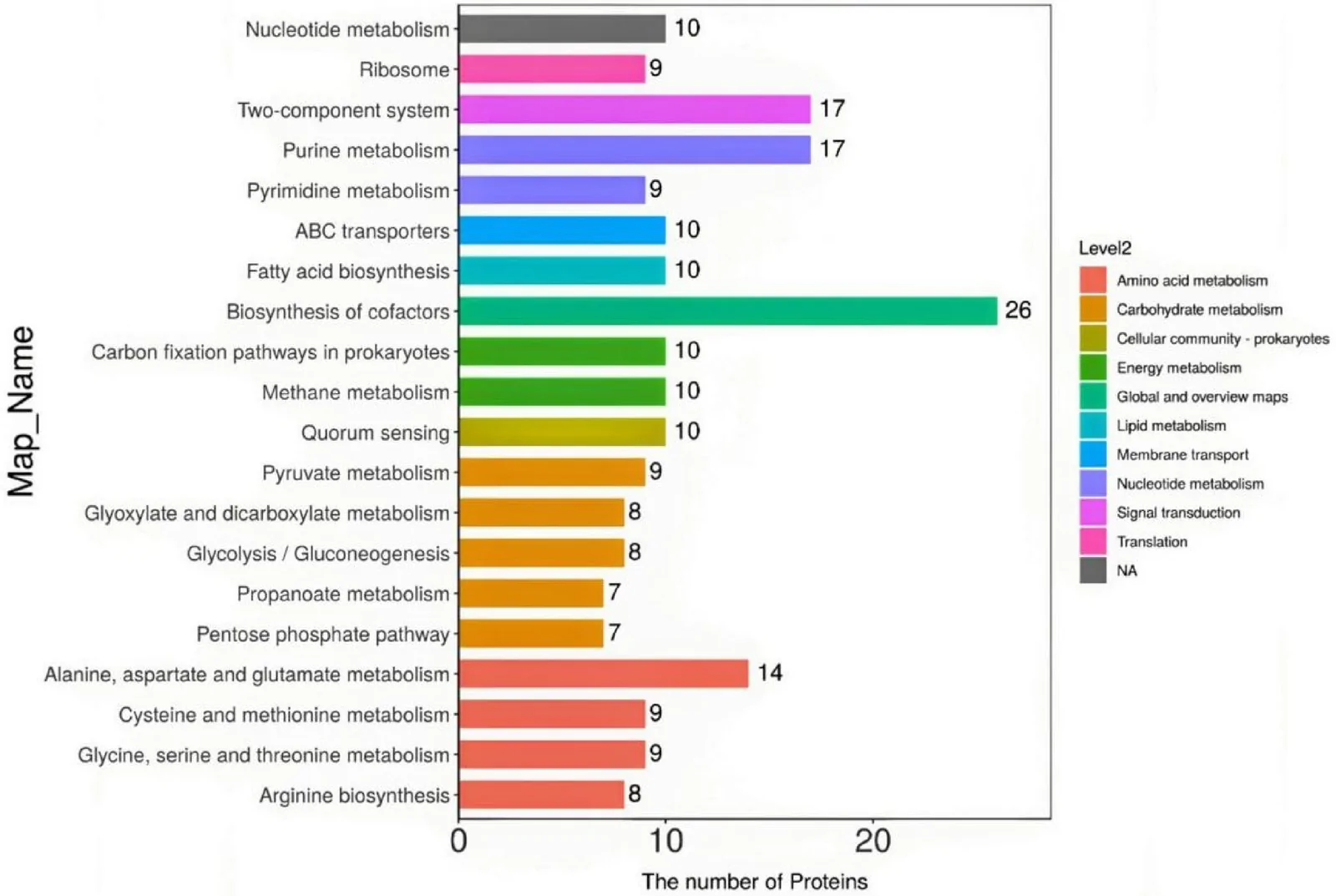
KEGG pathway statistical chart. KEGG, Kyoto Encyclopedia of Genes and Genomes.

**Figure 22:**
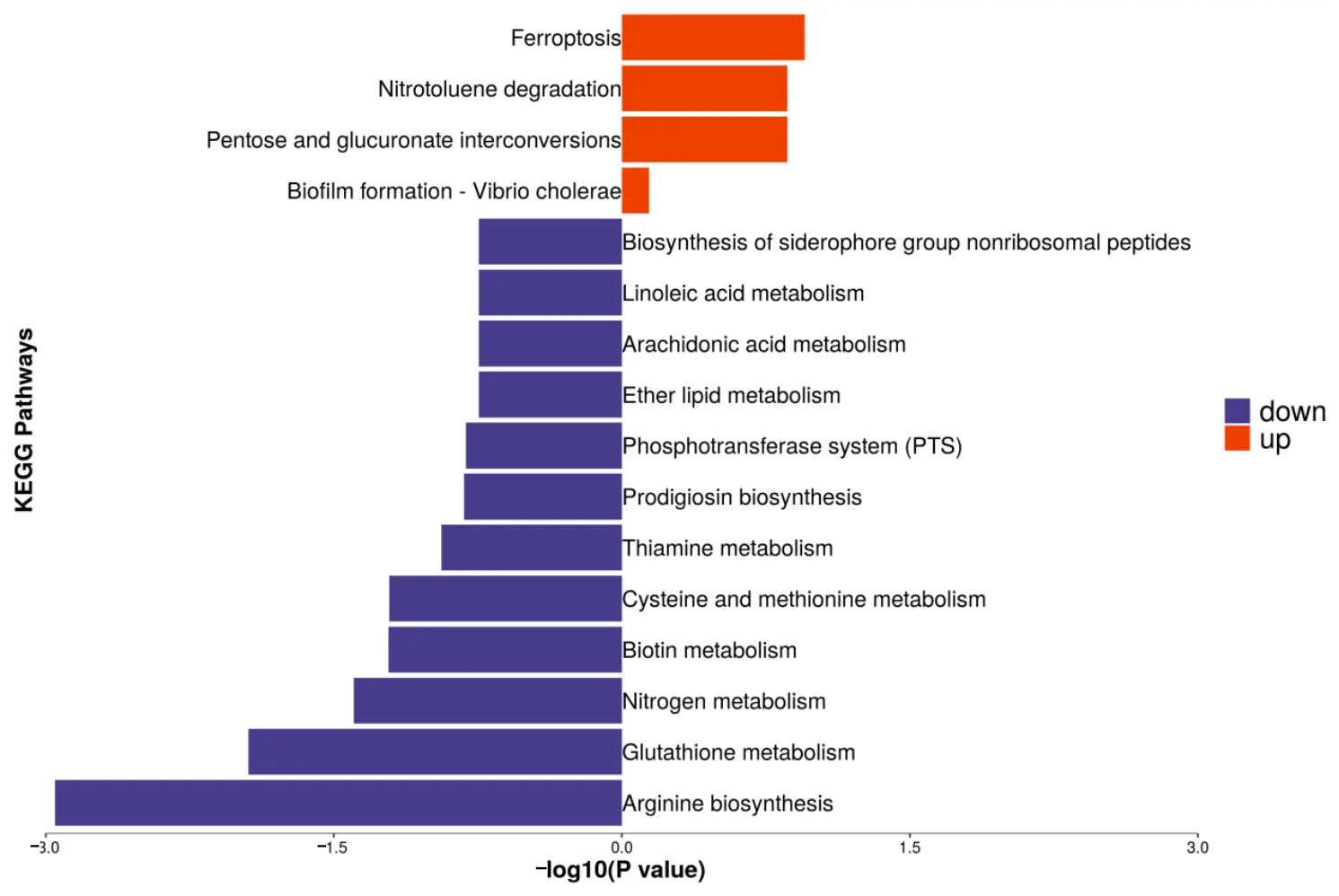
Statistical map of differential protein enrichment pathways.

Differentially expressed proteins were analyzed using KEGG pathway annotation, and protein counts were assessed at the secondary pathway level [18]. As shown in Figure 21 and Figure 22, the top 20 enriched KEGG pathways were identified. Pathways with the highest numbers of associated proteins included cofactor biosynthesis, nucleotide metabolism, ABC transporters, purine metabolism, fatty acid biosynthesis, quorum sensing, carbon fixation pathways in prokaryotes, alanine/aspartate/glutamate metabolism, methane metabolism, and two-component systems.

KEGG enrichment analysis further revealed that upregulated proteins were primarily associated with four pathways: ferroptosis, nitrobenzene degradation, pentose and glucuronate interconversions, and biofilm formation ability in *Vibrio cholerae*. In contrast, downregulated proteins were mainly enriched in 12 pathways, including arginine biosynthesis, glutathione metabolism, nitrogen metabolism, and biotin metabolism. Among these, proteins involved in arginine biosynthesis exhibited the greatest degree of downregulation, followed by those associated with glutathione metabolism and nitrogen metabolism. Conversely, proteins involved in biofilm formation ability in *V. cholerae* showed the smallest fold changes among all differentially expressed proteins.

#### 3.8.7. Protein–Protein Interaction Network Analysis

One of the primary mechanisms by which proteins exert their functions is through interactions with other proteins, either by mediating specific pathways or forming complexes to achieve biological regulation [19].

**Figure 23:**
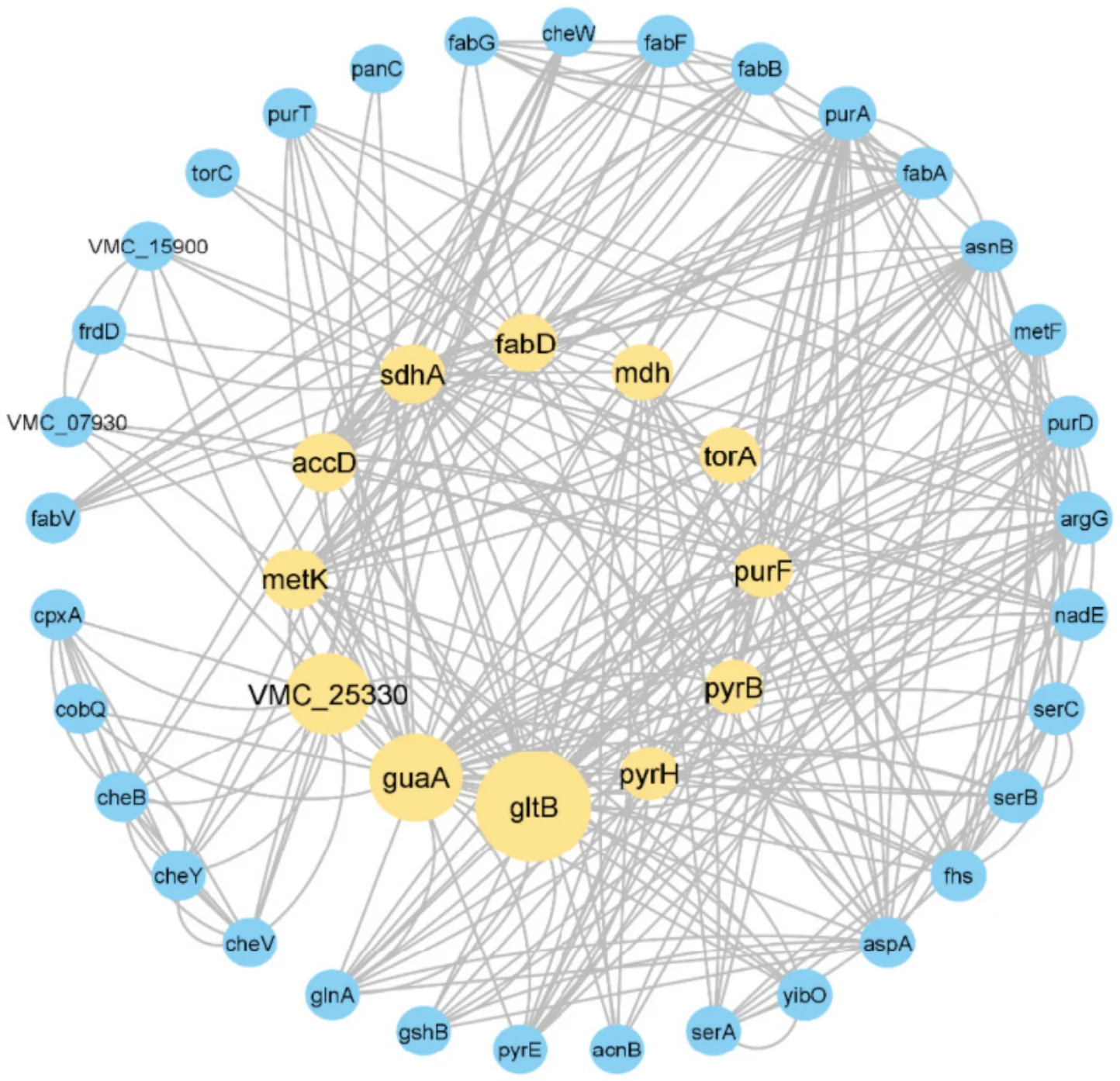
Differential protein interaction network diagram.

Based on protein–protein interaction data from the STRING database, a protein–protein interaction (PPI) network was constructed for the differentially expressed proteins using Cytoscape software v3.10.4 [20]. As shown in Figure 23, proteins mapped to the top three enriched KEGG pathways (cofactor biosynthesis, two-component system, and purine metabolism) were selected to construct the PPI network. In the network, the upregulated proteins included VMC_07930, TorA, and TorC, whereas the remaining proteins were downregulated.

### 3.9. Antibacterial Susceptibility Assay

**Figure 24:**
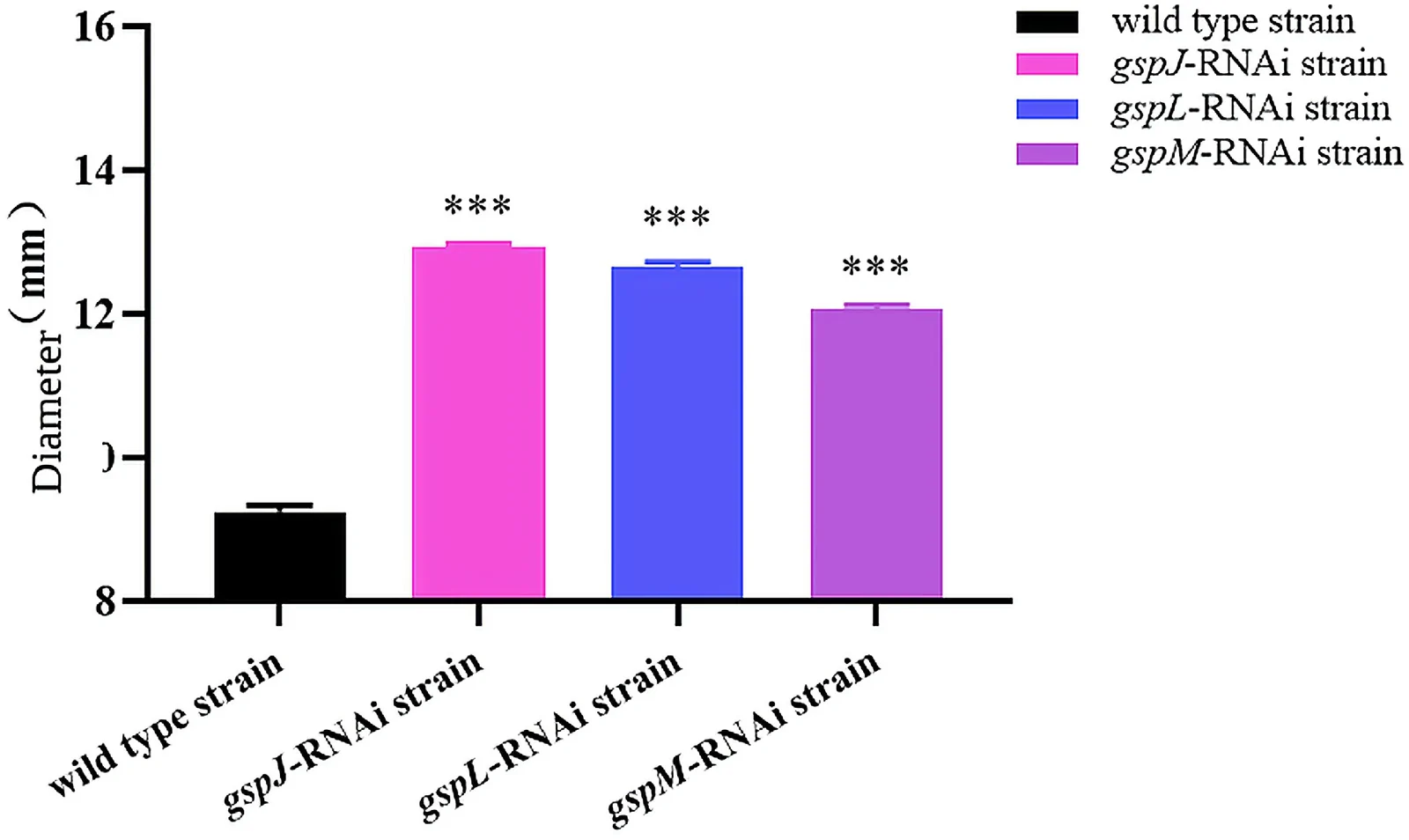
Antibacterial test results of wild-type *V. alginolyticus*, *gspJ*-RNAi strain, *gspL*-RNAi strain, and *gspM*-RNAi strain. *** *p* < 0.001.

The wild-type *V. alginolyticus* and *gspJ*-RNAi, *gspL*-RNAi, and *gspM*-RNAi strains were exposed to 70 mg/mL *S. baicalensis* extract, and inhibition zone diameters were measured. As shown in Figure 24, compared to the wild-type *V. alginolyticus*, the inhibition zone diameter increased 1.40-fold for the *gspJ*-RNAi strain, 1.30-fold for the *gspL*-RNAi strain, and 1.37-fold for the *gspM*-RNAi strain, showing that silencing of *gspJ*, *gspL*, and *gspM* markedly affects the antimicrobial susceptibility of *V. alginolyticus*.

## 4. Discussion

*V. alginolyticus is a major pathogen in aquaculture, and its pathogenicity is closely linked to the type II secretion system (T2SS) for secreting virulence effectors* [3,4]. Previous studies have established the importance of the T2SS in the secretion of extracellular proteins and in the virulence of pathogenic Vibrio species, but the individual contributions of specific structural components remain less well defined in *V. alginolyticus*. The individual contributions of core structural genes *gspJ*, *gspL*, and *gspM* remain uncharacterized. In this study, we constructed *gspJ*-RNAi, *gspL*-RNAi, and *gspM*-RNAi strains. We functionally characterized three T2SS-associated components, GspJ, GspL, and GspM, by integrating structural prediction, virulence-associated phenotyping, environmental response analysis, in vivo infection, and proteomic profiling. Our findings indicate that all three components contribute to multiple virulence-associated traits, while *gspL* showed an additional association with in vivo pathogenicity and broader changes in signaling, transport, and metabolic pathways.

The predicted structural features of the three proteins provide a possible framework for interpreting their distinct phenotypic effects. GspJ showed structural similarity to EpsJ, a minor pseudopilin associated with the T2SS pseudopilus. Minor pseudopilins are involved in the assembly and function of the secretion pseudopilus and may therefore influence the efficiency of extracellular protein transport. Consistent with this structural role, silencing *gspJ* markedly affected biofilm formation, motility, adhesion, and chemotaxis in the present study. GspL, in contrast, contains a large cytoplasmic region and showed structural similarity to the GspL domain that interacts with the ATPase GspE. This location places GspL at an important interface between ATP-dependent energy generation and the inner-membrane secretion machinery, which may help explain why *gspL* silencing was associated not only with virulence-related phenotypes but also with broader proteomic changes. GspM was predicted to be a membrane-associated protein and showed structural similarity to PilO-like components of type IV pilus systems, consistent with a potential role in stabilizing or organizing the inner-membrane platform. Together, these structural differences may contribute to the distinct phenotypic responses observed after silencing each gene.

Motility was differentially affected: gspL silencing enhanced motility by 38.49%, while *gspJ* and *gspM* silencing reduced it. It is speculated that *gspL* regulates motility via negative feedback. Previous studies showed that gspL absence significantly alters virulence gene transcription (e.g., luxS upregulation) and enhances virulence [21], suggesting that losing GspL relieves gene inhibition and remodels the regulatory network. Enhanced motility may thus be an incidental phenotype of this global compensation. Under pH stress treatment, *gspM* expression decreased dramatically while *gspJ* and *gspL* were up-regulated. Regarding the “gatekeeper” role of GspM, the downregulation of *gspM* expression may indicate that under pH stress, the bacteria actively “close” the assembly of the secretion channel to reduce unnecessary energy consumption or prevent erroneous secretion. This suggests that *gspL* may participate in a regulatory circuit where T2SS dysfunction triggers compensatory resource redistribution toward flagellar machinery, a common adaptive response to stress. These results are in agreement with our findings, in which silencing the *pstS* or *pstB* gene in *V. alginolyticus* dramatically reduced adhesion (73.57–79.61%) and biofilm formation ability (24.99–84.01%). These results align with the T2SS’s canonical function, as secretion of adhesion factors and extracellular matrix components is essential for colonization and biofilm integrity [3,22]. The observed impairments likely stem from disrupted effector protein secretion.

To elucidate molecular mechanisms, we performed label-free proteomics on the *gspL*-RNAi strain; the analysis provided additional evidence that the effects of *gspL* silencing extend beyond the secretion apparatus itself, identifying 189 differentially expressed proteins (144 downregulated, 45 upregulated). These were significantly enriched in pathways related to two-component signaling, ABC transport, and amino acid metabolism, indicating that loss of *gspL* causes systemic regulatory perturbation rather than isolated defects.

Notably, proteins related to the two-component signal system (TCS), particularly response regulator receiver domains, were significantly altered. Given that TCS is central to environmental sensing and virulence regulation [23], these changes suggest an association between GspL-dependent T2SS function and broader cellular regulatory networks. While TCS can regulate T2SS expression [23], our results indicate that T2SS integrity, via *gspL*, consequently modulates TCS protein expression, coordinating host adaptation and virulence output.

Similarly, reduced representation of ABC transport and metabolic pathways may impair nutrient acquisition, stress adaptation, and the metabolic capacity required for host colonization [14]. ABC transporters and amino acid metabolism pathways, including alanine, aspartate, and glutamate metabolism, were significantly downregulated [16]; their suppression likely limits bacterial survival in nutrient-poor host environments. Concurrent metabolic remodeling would further constrain energy and precursors available for virulence factor synthesis and biofilm construction [24,25].

A notable contribution of the present study is the integration of gene-specific phenotyping with in vivo and proteomic analyses. We establish *gspL* not merely as a structural component but as a central hub linking signal transduction, metabolism, and pathogenicity [3,4]. Rather than showing only that disruption of T2SS-associated genes alters virulence-related phenotypes, our results suggest that GspL is associated with broader changes in signaling, transport, and metabolic pathways [25,26]. This extends the functional interpretation of GspL beyond its established structural role within the T2SS and provides a basis for investigating how secretion system components may interact with wider cellular regulatory networks in *V. alginolyticus* [27].

At the applied level, this work suggests two anti-virulence strategies for aquaculture. First, *gspL* itself is a promising drug target; inhibiting it could disrupt T2SS function and regulatory homeostasis, attenuating virulence without the strong selective pressure of bactericidal agents [28,29]. Second, our previous finding that *S. baicalensis* extract downregulates these genes, combined with reports that organic acids suppress T2SS expression [30], highlights the potential of screening natural products, particularly traditional Chinese medicines, for T2SS-specific inhibitors [9]. Quorum sensing inhibitors could also serve as auxiliary targets [16].

In summary, this study integrates genetics, phenomics, and proteomics to systematically elucidate the central roles of *gspJ*, *gspL*, and *gspM* in regulating adhesion, biofilm formation ability, and motility. Crucially, it reveals for the first time that *gspL* globally regulates pathogenicity by modulating TCS and basic metabolic networks. These findings provide both theoretical insights into *V. alginolyticus* pathogenesis and novel targets for developing green, effective, and low-resistance strategies to control vibriosis in aquaculture. The distinct transcriptional responses of *gspJ*, *gspL*, and *gspM* to pH and temperature further suggest that these T2SS-associated components are not regulated uniformly under environmental stress. In particular, *gspJ* showed strong induction under alkaline conditions, whereas *gspL* responded most strongly under acidic conditions and *gspM* was generally suppressed across non-neutral pH conditions. Temperature also produced gene-specific expression patterns. These differences suggest that the three components may be subject to distinct regulatory controls despite functioning within the same secretion system. Because environmental conditions such as pH and temperature are closely linked to bacterial physiology and virulence in aquatic environments, such differential regulation may allow *V. alginolyticus* to adjust T2SS activity under changing external conditions.

## 5. Conclusions

This study systematically investigated the roles of the T2SS-associated components GspJ, GspL, and GspM in the pathogenicity of *V. alginolyticus*. Silencing of these genes altered multiple virulence-associated phenotypes, including biofilm formation, motility, adhesion, and chemotaxis, indicating that all three components contribute to bacterial functions related to host colonization and pathogenicity. Among them, *gspL* showed a particularly important role in vivo, as its silencing reduced host mortality and bacterial colonization in *E. coioides’s* liver and spleen. Proteomic analysis further showed that *gspL* silencing was associated with changes in proteins associated with two-component signaling, ABC transport systems, and amino acid metabolism, suggesting that GspL may be functionally linked to broader cellular regulatory and metabolic processes in addition to its structural role in the T2SS. The distinct transcriptional responses of *gspJ*, *gspL*, and *gspM* to changes in pH and temperature further indicate that these genes are differentially regulated under environmental stress. Together, these findings provide new functional evidence for the involvement of GspJ, GspL, and GspM in *V. alginolyticus* virulence and expand the current understanding of how individual T2SS components may contribute to pathogenicity, and provide a basis for exploring these components as potential targets for future anti-virulence strategies in aquaculture.

## Figures and Tables

**Table 1:** Target genes qRT-PCR primers.

Primer Name	Sequence (5′-3′)
*gyrB*	F: TTGATTTACACTCCTCGCCTAAC R: CAGCCAACTTGCCGTACTCT
*gspJ*	F: ATGTTTGCTCGCTTAGGATG R: CCACTGGCGTGTCAGGGTAG
*gspL*	F: TTGTCGATGCTTCTTGGTTA R: CAAATTGACTTTGCTTTCTGTA
*gspM*	F: TTCTTCTTCAGCCCGTCGTT R: CAATACCCTGTTGCTCTTTCA

**Table 2:** shRNA sequences.

shRNA	Sequence (5′-3′)
*gspJ-RNAi-159*	F: GATCCGCGAACTCAGCGCTTAAATGATTCAAGAGATCATTTAAGCGCTGAGTTCGCTTTTTTGCATG R: CAAAAAAGCGAACTCAGCGCTTAAATGATCTCTTGAATCATTTAAGCGCTGAGTTCGCG
*gspJ-RNAi-315*	F: GATCCGCTAATGTTTGCTCGCTTAGGTTCAAGAGACCTAAGCGAGCAAACATTAGCTTTTTTGCATG R: CAAAAAAGCTAATGTTTGCTCGCTTAGGTCTCTTGAACCTAAGCGAGCAAACATTAGCG
*gspJ-RNAi-562*	F: GATCCGCTAATGTTTGCTCGCTTAGGTTCAAGAGAAGTAAGGATCACAGAAACCGCTTTTTTGCATG R: CAAAAAAGCGGTTTCTGTGATCCTTACTTCTCTTGAACCTAAGCGAGCAAACATTAGCG
*gspL-RNAi-113*	F: GATCCGGGAACACTTAGATGAGTTGATTCAAGAGATCAACTCATCTAAGTGTTCCCTTTTTTGCATG R: CAAAAAAGGGAACACTTAGATGAGTTGATCTCTTGAATCAACTCATCTAAGTGTTCCCG
*gspL-RNAi-346*	F: GATCCGCTTGGATTCAGACCGTATTGTTCAAGAGACAATACGGTCTGAATCCAAGCTTTTTTGCATG R: CAAAAAAGCTTGGATTCAGACCGTATTGTCTCTTGAACAATACGGTCTGAATCCAAGCG
*gspL-RNAi-762*	F: GATCCGGGCGTGCTTATCGTTGTTATTTCAAGAGAATAACAACGATAAGCACGCCCTTTTTTGCATG R: CAAAAAAGGGCGTGCTTATCGTTGTTATTCTCTTGAAATAACAACGATAAGCACGCCCG
*gspM-RNAi-148*	F: GATCCGCTCAATCACGTATTCAAAGTTTCAAGAGAACTTTGAATACGTGATTGAGCTTTTTTGCATG R: CAAAAAAGCTCAATCACGTATTCAAAGTTCTCTTGAAACTTTGAATACGTGATTGAGCG
*gspM-RNAi-344*	F: GATCCGGATCAAACCACTCGCGTTTATTCAAGAGATAAACGCGAGTGGTTTGATCCTTTTTTGCATG R: CAAAAAAGGATCAAACCACTCGCGTTTATCTCTTGAATAAACGCGAGTGGTTTGATCCG
*gspM-RNAi-441*	F: GATCCGGATCAAGCGGGCATGATTGATTCAAGAGATCAATCATGCCCGCTTGATCCTTTTTTGCATG R: CAAAAAAGGATCAAGCGGGCATGATTGATCTCTTGAATCAATCATGCCCGCTTGATCCG

## Data Availability

Raw experimental data are not publicly archived due to laboratory storage regulations but are accessible from the corresponding author upon reasonable request.
